# Genomic analysis of worldwide sheep breeds reveals *PDGFD* as a major target of fat-tail selection in sheep

**DOI:** 10.1186/s12864-020-07210-9

**Published:** 2020-11-17

**Authors:** Kunzhe Dong, Min Yang, Jiangang Han, Qing Ma, Jilong Han, Ziyi Song, Cuicheng Luosang, Neena Amatya Gorkhali, Bohui Yang, Xiaohong He, Yuehui Ma, Lin Jiang

**Affiliations:** 1 National Germplasm Center of Domestic Animal Resources, Institute of Animal Sciences, Chinese Academy of Agricultural Sciences (CAAS), No. 2 Yuanmingyuan West Road, Beijing, 100193 China; 2 Key Laboratory of Animal (Poultry) Genetics Breeding and Reproduction, Ministry of Agriculture and Rural Affairs, CAAS, Beijing, 100193 China; 3 Present address: Department of Pharmacology and Toxicology, Medical College of Georgia, Augusta University, Augusta, GA 30912 USA; 4 College of Animal Science and Technology, Shihezi University, Shihezi, 832000 China; 5 Research Center of Grass and Livestock, Ningxia Academy of Agriculture and Forestry Sciences, Yinchuan, 750002 China; 6 College of Animal Science and Technology, Guangxi University, Nanning, 530004 Guangxi China; 7 Research Institute of Animal Science, Tibet Academy of Agricultural and Animal Husbandry Sciences, Lhasa, 850000 China; 8 Lanzhou Institute of Husbandry and Pharmaceutical Sciences, Chinese Academy of Agricultural Sciences (CAAS), Lanzhou, 730050 China

**Keywords:** *PDGFD*, Fat-tailed sheep, Genomic scan, Fat deposit, Adipogenesis

## Abstract

**Background:**

Fat tail is a unique trait in sheep acquired during domestication. Several genomic analyses have been conducted in sheep breeds from limited geographic origins to identify the genetic factors underlying this trait. Nevertheless, these studies obtained different candidates. The results of these regional studies were easily biased by the breed structures.

**Results:**

To minimize the bias and distinguish the true candidates, we used an extended data set of 968 sheep representing 18 fat-tailed breeds and 14 thin-tailed breeds from around the world, and integrated two statistical tests to detect selection signatures, including Genetic Fixation Index (*F*_*ST*_) and difference of derived allele frequency (ΔDAF). The results showed that *platelet derived growth factor D* (*PDGFD*) exhibited the highest genetic differentiation between fat- and thin-tailed sheep breeds. Analysis of sequence variation identified that a 6.8-kb region within the first intron of *PDGFD* is likely the target of positive selection and contains regulatory mutation(s) in fat-tailed sheep. Histological and gene expression analyses demonstrated that *PDGFD* expression is associated with maturation and hemostasis of adipocytes. Further retrospective analysis of public transcriptomic datasets revealed that *PDGFD* expression is down-regulated during adipogenesis in both human and mouse, and is higher in fat tissues of obese individuals than that in lean individuals.

**Conclusions:**

These results reveal that *PDGFD* is the predominant factor for the fat tail phenotype in sheep by contributing to adiopogenesis and maintaining the hemostasis of mature adipocytes. This study provides insights into the selection of fat-tailed sheep and has important application to animal breeding, as well as obesity-related human diseases.

**Supplementary Information:**

The online version contains supplementary material available at 10.1186/s12864-020-07210-9.

## Background

Following domestication in the Fertile Crescent approximately 8000–9000 years ago [1], sheep spread over a wide geographical range worldwide. To adapt to nutrient-poor diets and extreme environments, sheep develop a substantial variation in many phenotypic traits [2]. One of the main morphological changes from wild ancestors is the lengthening of the tail and distinct patterns of tail fat deposition. It is believed that fat-tailed sheep were selected in response to the steppe and desert conditions in central Asia from 3, 000 BCE (Before Common Era) [3], which is several thousand years after the domestication of its thin-tailed ancestor, and then spread east into north China and west into South Africa.

Today, the fat-tail phenotype is largely undesirable for both producers and consumers, partially because that fat tails have a significant influence on fat deposition in other parts of the body [4], mating and normal locomotion of the animal [5]. Additionally, the consumers in many instances show an increasing preference for lean meat. Genetic improvement is a more attractive strategy to reduce tail size than traditional husbandry practices, like tail docking, as it is reliable, long lasting and may improve animal welfare. For this purpose, finding the genes underlying the fat tail phenotype is the first and most important thing.

Several efforts have been made aiming to identify genes or genomic regions associated with fat tail phenotype by genome-wide scans [6–11]. However, the results of these studies remain inconclusive, with almost no consensus on their implications. This is quite surprising, as all the fat-tailed sheep seem to be of same origin with relatively short history and thus are expected to have the similar genetic basis underlying the trait. Additionally, these previous studies suffered several limitations that hinder the search for valid candidates. First, they investigated sheep populations from relatively few areas, which may identify the wrong candidate loci that were selected by other confounding factors such as geographic isolation. Second, these studies mostly applied allele frequency-based methods, such as Genetic Fixation Index (*F*_*ST*_) method, to identify candidate genes. *F*_*ST*_-based approach is widely used to identify the region with high divergence between populations; however, it lacks the power of detecting the direction of selection. To clarify this, we performed a comprehensive genomic analysis of 18 thin-tailed and 14 fat-tailed sheep breeds from around the world. We integrated two selection tests, including *F*_*ST*_ and the difference of derived allele frequency (ΔDAF) (referred to as DAF_Fat-tailed sheep_ – DAF_Thin-tailed sheep_), to identify positively selected genes specifically in fat-tailed sheep. Whole genome-sequencing data of the candidate genes were further analyzed to detect the potentially casual variants. Additionally, histological and gene expression analysis of sheep tail tissues were carried out to understand the association of the expression of the most predominant candidate gene *PDGFD* with fat deposition in sheep tail during embryonic development. Finally, re-analysis of public transcriptomic datasets were performed to examine the change of *PDGFD* expression during adipogenesis in human and mouse, as well as between the fat tissues of obese and lean individuals.

## Results

### Identification of genes underlying fat tail in sheep

**Fig. 1:**
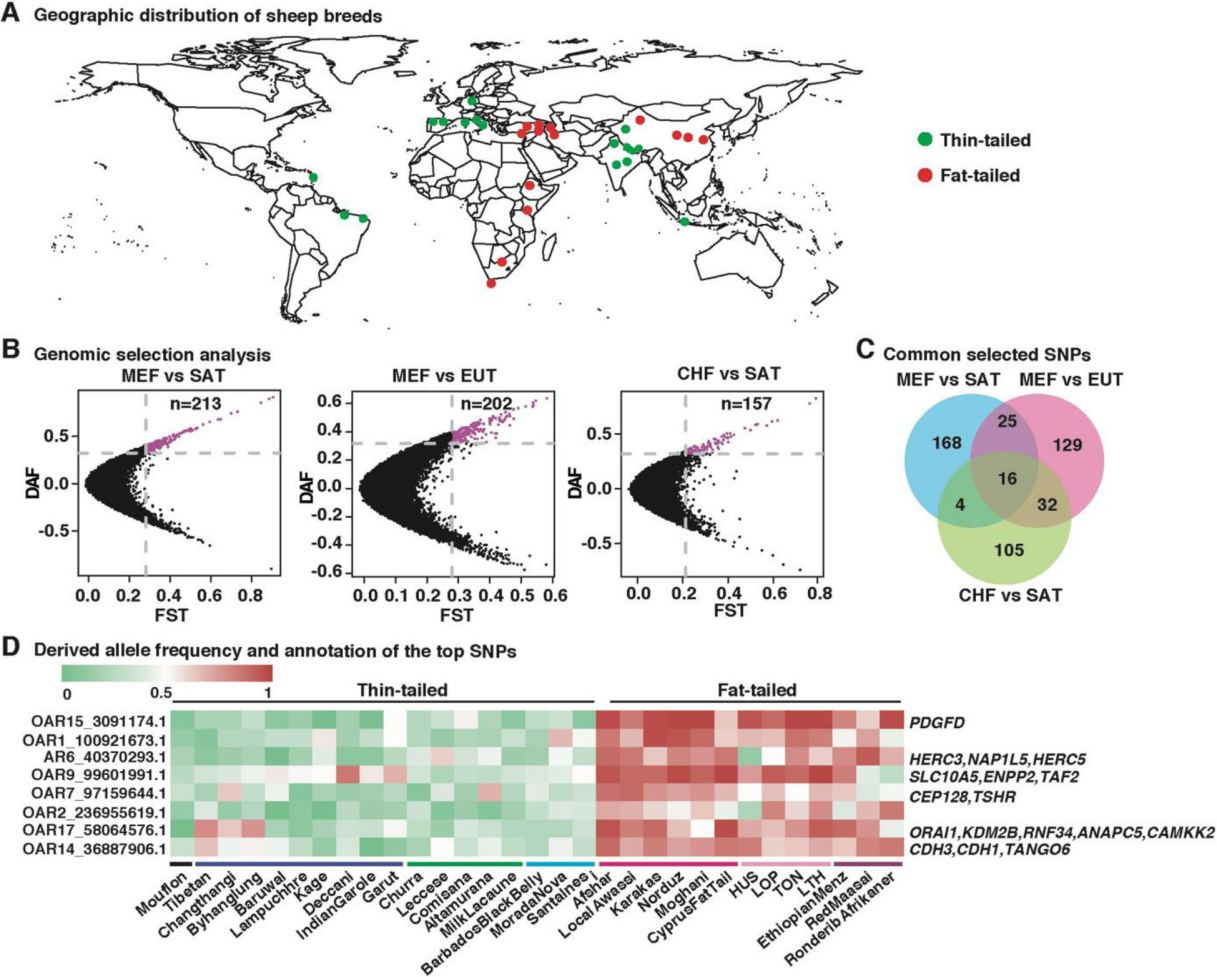
Identification of positively selected loci in fat-tailed sheep. **a** The geographic distribution of the sheep breeds used in this study, each of which is represented by a dot on the world map. The map was generated with R package “rworldmap” (https://cran.r-project.org/web/packages/rworldmap/). **b** The number of positively selected loci identified in three different group-pair comparisons. MEF: Middle East fat-tailed sheep from Middle East; SAT: South Asian thin-tailed sheep; EUT: European thin-tailed sheep; CHF: Chinese fat-tailed sheep. **c** The number of positively selected loci identified in all the three group-pair comparisons. **d** The derived allele frequency (DAF) of the 16 positively selected loci identified in all three group-pair comparisons in all the studied sheep breeds. SNPs were sorted according to the average value of *F*_*ST*_ and ΔDAF among the three group-pair comparisons indicated in Fig. 1B from high to low. The important annotated genes of each SNP were labeled in the right

SNP Beadchip data for a total of 968 individual sheep was collected and used in this study. These samples contained eight wild Mouflon sheep individuals, 18 thin-tailed sheep breeds and 14 representative fat-tailed sheep breeds from different countries or regions within countries (Fig. 1a**;** Additional file 1**: Table S1**). The geographic distributions of the sheep breeds in this report include South Asia, East Asia, Middle East, Europe, Africa, North America, and South America (Fig. 1a**;** Additional file 1**: Table S1**). After a series of quality control filters, a total of 45,337 SNPs and 828 individuals from 30 diverse sheep breeds worldwide were retained for further analysis.

Because the wild ancestor of domestic sheep, the Mouflon sheep, is thin-tailed, modern thin-tailed sheep are expected to keep initial allele state while fat-tailed sheep exhibit derived alleles at the loci related to fat tail phenotype. Under this scenario, we integrated two statistical measures, the *F*_*ST*_ and the ΔDAF, to increase the power of identifying genomic loci specifically selected in fat-tailed sheep breeds. For comparison with previous studies, we separately performed *F*_*ST*_ and ΔDAF analysis in three group-pair comparisons, including Middle East fat-tailed sheep (MEF) vs South Asian thin-tailed sheep (SAT), MEF vs European thin-tailed sheep (EUT), and Chinese fat-tailed sheep (CHF) vs SAT. In total, 213, 202 and 157 positively selected SNPs were identified by both *F*_*ST*_ and ΔDAF methods in the three comparisons, respectively (Fig. 1b) (See Additional file 2-4**: Tables S2-S4** for the full significant SNP list). Among them, 16 loci were common to all the three group-pair comparisons (Fig. 1c).

Eight out of the 16 common significant SNPs were further ruled out from our candidate list because they have DAF > 0.5 (or DAF < 0.5) in more than 30% of the thin-tailed (or fat-tailed) sheep breeds, after examining the DAF of these SNPs in additional three American thin-tailed sheep breeds and three African fat-tailed sheep breeds (See Methods) (Additional file 5**: Fig. S1**). The remaining eight SNPs are highly diverged between thin- and fat-tailed sheep, and keep ancestral allele in most thin-tailed sheep breeds while derived allele in most fat-tailed sheep breeds worldwide (Fig. 1d), indicative of promising candidates associated with fat tail phenotype. Principle Component Analysis (PCA) and phylogenetic tree analysis using these eight SNPs showed clearly separated clades between thin- and fat-tailed sheep, which is quite distinct from the results obtained based on genome-wide variants showing geographic clustering (Additional file 6**: Fig. S2**) and further supported the strong divergence of these loci. Gene annotation of 150 kb-long genomic region surrounding the eight SNPs revealed 24 genes and *PDGFD* corresponding SNP OAR15_3091174 among them that is located on chromosome 15 was most highly differentiated (Fig. 1d).

### Analysis of sequence variations of *PDGFD* region

**Fig. 2:**
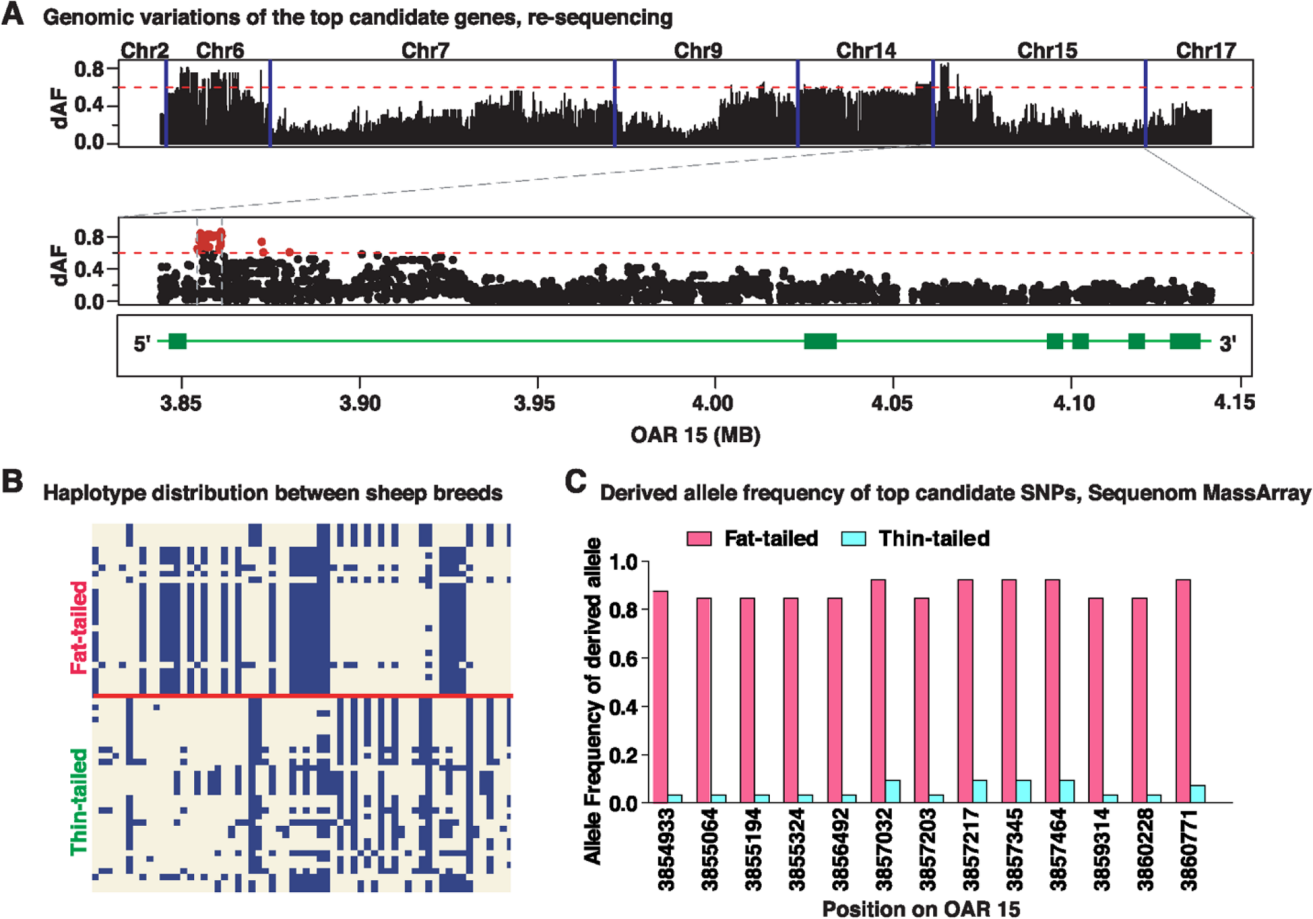
Analysis of sequence variations of *PDGFD* gene. **a** The distribution of the absolute difference of allele frequency ΔAF of sequence variation within the 24 identified genes with selection signatures between 13 fat-tailed sheep individuals and 16 thin-tailed sheep individuals from different regions. The sequence variations were downloaded from NextGene project. The red dashed line indicates the threshold value (ΔAF > 0.6). The bottom panel shows the distribution of the absolute difference of allele frequency ΔAF of sequence variation within *PDGFD* gene. The red dashed line indicates the threshold value (ΔAF > 0.6) and red dots represents SNPs with ΔAF > 0.9. **b** Haplotype compassion between thin-tailed and fat-tailed sheep obtained based on all the variations within the highly differentiated 6.8-kb genomic interval (Chr15:3,854,063-3,860,894 bp). Each column is a polymorphic genomic location (122 in total), each row is a phased haplotype (16 thin-tailed sheep and 13 fat-tailed sheep individuals), and alternative alleles are labelled in blue. **c** Derived allele frequency of the 13 top candidate SNPs in additional fat-tailed sheep (Chinese Tan sheep) and thin-tailed sheep (Chinese Tibetan sheep) obtained by Sequenom MassARRAY

To better investigate these selected genes, we extracted their genome sequence variants of 16 thin-tailed and 13 fat-tailed individual sheep with diverse geographic origins from NextGen project (Additional file 7**: Table S5**). A total of 17,124 SNPs were identified in the selected regions among these individual sheep (MAF > 0.05). We calculated the absolute difference of allele frequency (ΔAF) of each SNP and confirmed that the *PDGFD* gene is the most divergent locus between fat- and thin-tailed sheep (Fig. 2a), again implying that *PDGFD* is likely the most promising candidate for the sheep tail divergence. PDGFD is one of the platelet-derived growth factor (PDGF) family members, which play an important role in regulation of adipocyte development and function. The expression pattern across multiple issues obtained from Human Protein Atlas database (http://www.proteinatlas.org/) showed that *PDGFD* is relatively more abundantly expressed in pancreas, pituitary, ovary and adipose tissue than that in other tissues (Additional file 8**: Fig. S3**). These already known involvements of *PDGFD* in lipid metabolism provide further support for the hypothesis that this gene is an ideal candidate for fat tail in sheep.

Furthermore, ΔAF is noted to be particularly elevated in a 6.8-kb region (Chr15: 3,854,063 - 3,860,894 bp) in *PDGFD* gene containing 51 most differentiated SNPs (ΔAF > 0.6) (Additional file 9**: Table S6**). Haplotype comparison analysis using all the SNPs (*n* = 122) within this region revealed a consistent differentiation between sheep breeds with different tail types (Fig. 2b). Therefore, it is likely that this 6.8-kb region is the target of positive selection for fat tail and is the best candidate region for the functional mutation(s). This region is located at the first intron of *PDGFD* (Fig. 2b, bottom), suggesting that the mutations causing the association with the fat-tail phenotype are regulatory. We next genotyped a total of 13 SNPs that exhibit highest ΔAF value (> 0.8) between thin- and fat-tailed sheep and large derived allele frequency in fat-tailed sheep (> 0.8) based on genome re-sequencing results, using Sequenom MassARRAY in an expanded cohort containing 200 Tibetan sheep (Thin-tailed sheep) and 184 Tan sheep (fat-tailed sheep). This analysis confirmed that all these SNPs are highly divergent (Fig. 2c). Collectively, these observations strongly suggested that the 6.8-kb region as well as the 13 most highly differentiated SNPs identified here deserves further investigation for revealing the causative mutation(s).

In addition to *PDGFD*, several other selected genes identified in this study have known functions that are associated with lipid metabolism, such as *ENPP2*, *ANAPC5*, *RNF34*, *KDM2B*, *CAMKK2*, *TSHR*, and *CDH1*. The genetic differentiation for these genes was not as pronounced as for the *PDGFD* loci, implying that these loci likely contribute to the phenotypic difference with relatively small effects. We also examined the DAF distribution of the top candidate SNPs that were proposed in previous studies and the results showed that the majority of these SNPs are not consistently diverged between thin- and fat-tailed sheep populations (Additional file 10**: Fig. S4**).

### Histological analysis of sheep tail tissues during embryonic development

**Fig. 3:**
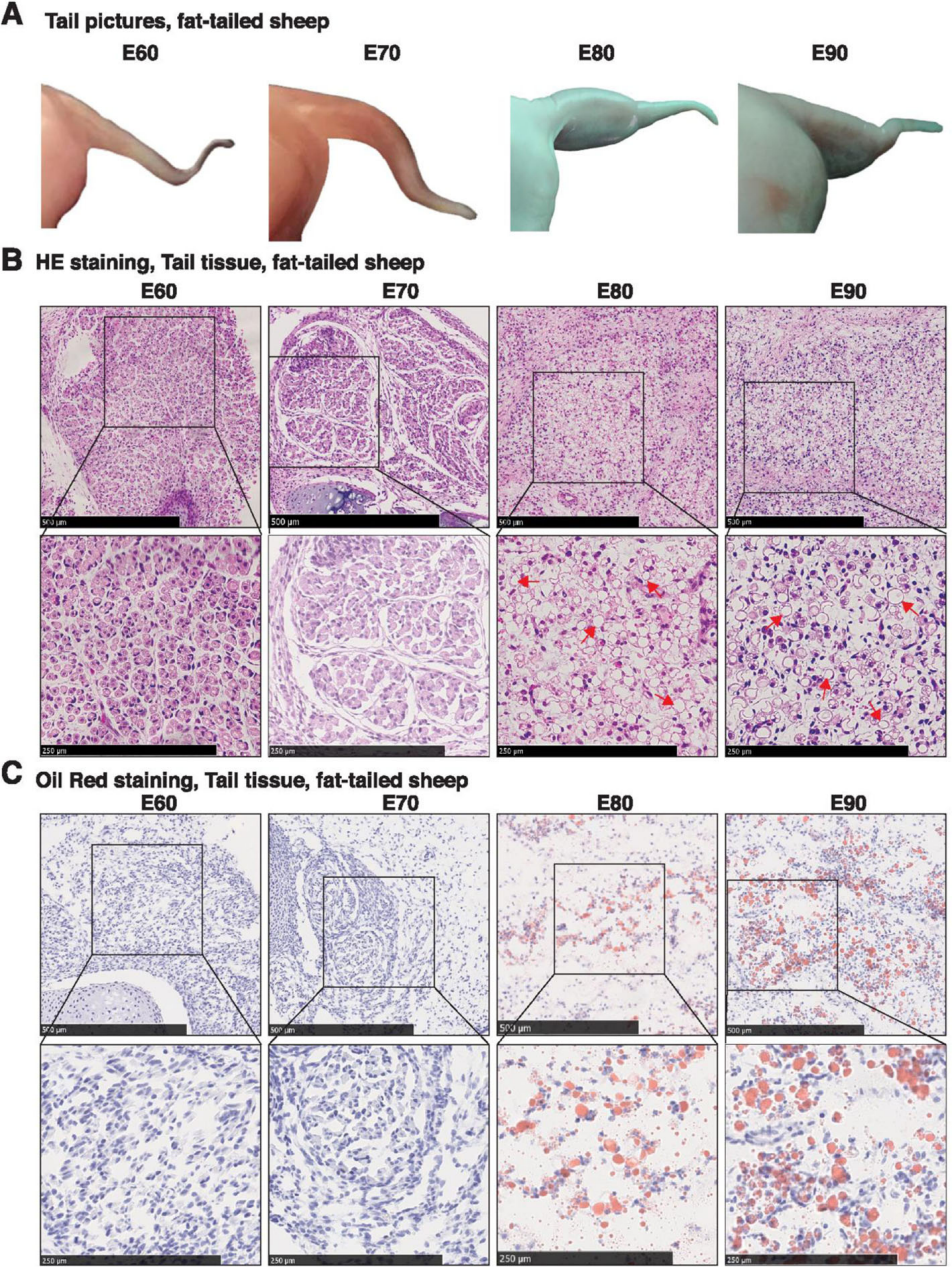
Histological analysis of sheep tail tissues during embryonic development. **a** Morphological changes of tail tissues from fat-tailed sheep at embryonic day 60 (E60), E70, E80 and E90. **b** Hematoxylin and Eosin (HE) staining for tail tissues of fat-tailed sheep at E60, E70, E80 and E90. The boxed areas are magnified on the bottom. Red arrows point to representative lipid drops. **c** Oil Red O staining for tail tissues of fat-tailed sheep at E60, E70, E80 and E90. The boxed areas are magnified on the bottom

To gain insight into tail fat deposition in fat-tailed sheep breeds, tail tissues from a fat-tailed Chinese sheep breed, namely Tan sheep, at four different embryonic time points, including embryonic day 60 (E60), E70, E80 and E90, were collected. It was observed that the tail is thinnest at E60 and E70 and, by E80 and E90, is much larger (Fig. 3a), indicating fat deposition. Further Hematoxylin and Eosin (HE) staining showed that lipid vacuole is absent in cells at E60 and E70, while accumulates in cells at E80 and E90, as evidenced by the observation that one large vacuole was present in the cell and the nucleus was packed in a corner (Fig. 3b), a typical feature of mature adipocytes. In line with this, Oil Red O staining which visualizes fat-containing droplets revealed that a large portion of cells at E80 and E90 showed distribution of lipid drops (Fig. 3c). These observations together suggested that committed preadioocytes are present in tail tissues at E60 and E70 which differentiate to mature adipocytes around E80 and E90.

### *PDGFD* expression is associated with adipogenesis and higher in adipose tissues of lean than that in fat individuals in different species

**Fig. 4:**
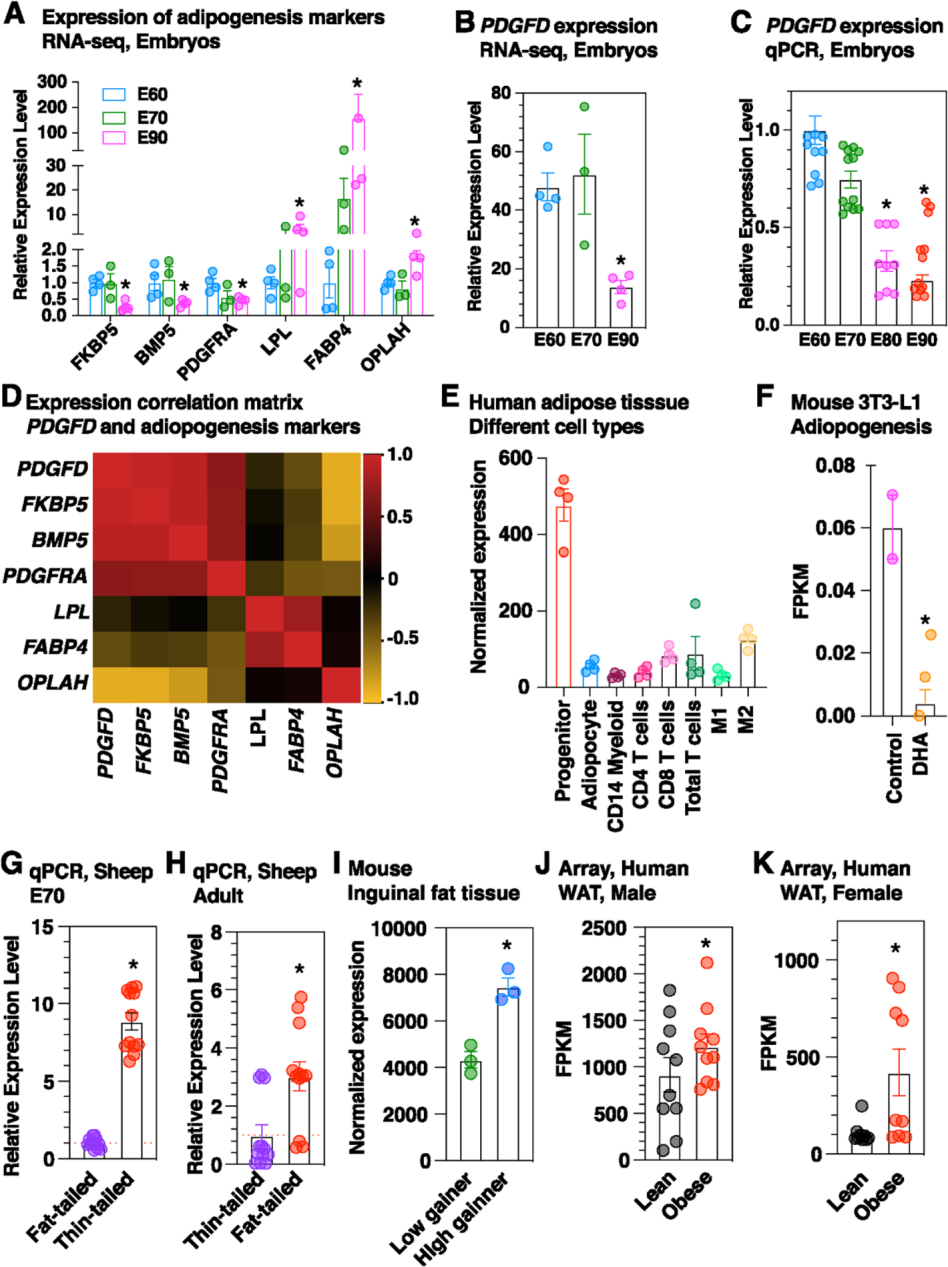
*PDGFD* expression is associated with adipogenesis and higher in adipose tissues of lean than that in fat individuals in different species. **a** Expression of several marker genes involved in adipogenesis in tail tissues of fat-tailed sheep at different stages of embryonic development revealed by RNA-seq. *PDGFD* expression in tail tissues of fat-tailed sheep at different stages of embryonic development revealed by **b** RNA-seq and **c** qPCR. **d** Heatmap showing the correlation matrix between expression of *PDGFD* and the indicated adipogenesis markers. **e** Expression of *PDGFD* in different cell fractions of human subcutaneous adipose tissues. Macrophages were subdivided into M1 and M2 (GSE100795). **f** Expression of *Pdgfd* during the adipogenesis of mouse 3 T3-L1 preadiopocytes induced by ω-3 fatty acid DHA revealed by RNA-seq (GSE118471). **g** *PDGFD* expression is higher in fat tissues of thin-tailed and fat-tailed sheep at E70 and **h** adult stage revealed by qPCR. *P < 0.05. **i** *Pdgfd* expression is higher in inguinal fat of high weight gaining mice than that of in low weight gaining mice revealed by microArray analysis (GSE4692). *P < 0.05 (*n* = 3). *PDGFD* expression is higher in adipocytes from lean than that from obese Indian individuals in both **j** male and **k** female subjects revealed by microarray (GSE2508)

As an initial step to elucidate the role of *PDGFD* in the development of fat tail in sheep, we performed gene expression analysis by RNA-seq for different embryonic stages. The results showed that the expression of some marker genes which are known to be down-regulated (*FKBP5*, *BMP5* and *PDGFRA*) and up-regulated (*LPL*, *FABP4* and *OPLAH*) during adipogenesis had no obvious difference between E60 and E70 while significantly decreased and increased at E80 compared to earlier stages, respectively (Fig. 4a), confirming the morphologic observation and histological results that cells in tail tissues of fat-tailed sheep undergo adipogenesis from E60/70 to E80/90. Interestingly, *PDGFD* expression was undistinguishable between E60 and E70, but was dramatically decreased at E80 (Fig. 4b). Subsequent Quantitative reverse transcription-PCR (qRT-PCR) analysis confirmed the large reduction of *PDGFD* expression at E80, followed with a continued but not significant decline at E90, as compared to that at E60 and E70 (Fig. 4c). Other candidate genes including *BMP2* which were proposed by several studies, exhibited no specific oscillation in expression mirroring the process of adipocyte maturation in tail tissues along with embryonic development (Additional file 11**: Fig. S5**). Gene expression correlation analysis revealed that *PDGFD* expression is highly positively correlated with markers enriched in preadipocytes and negatively correlated with markers up-regulated in mature adipocytes (Fig. 4d). Accordingly, retrospective analysis of public transcriptomic data sets revealed that *PDGFD* expression is higher in adipose progenitors than that in other cell types isolated from human white adipose tissues (Fig. 4e). Furthermore, its expression is dramatically reduced during the adipogenesis of mouse 3 T3-L1, although the abundance in 3 T3-L1 is relatively low (Fig. 4f). Taken together, these results suggest that *PDGFD* expression is enriched in preadipocytes and is down-regulated during adipogenesis.

Further qRT-PCR analysis showed that *PDGFD* expression is greater in tail tissues of fat-tailed sheep than that in thin-tailed sheep at both E70 and adult stages (Fig. 4g and h). Interestingly, re-analysis of public data sets revealed that *PDGFD* is also more abundantly expressed in adipose tissues of obese individuals than that of lean individuals in both mouse (Fig. 4i) and human (Fig. 4j and k), suggesting an evolutionarily conserved role of *PDGFD* involved in hemostasis of mature adipocytes.

## Discussion

To accurately map the candidate gene(s) underlying the fat tail phenotype of sheep, herein we comprehensively analyzed genomic variation data from a large cohort of sheep breeds with different tail types from around the world and integrated two different selection tests to detect genomic regions with signals of selection. We demonstrated that *PDGFD* gene locus, with known involvement in adipogenesis [12, 13], exhibited the highest genetic differentiation between fat- and thin-tailed sheep and further found that the potential causal mutations are located within regulatory region. In addition, we show that *PDGFD* expression is negatively associated with maturation of adipocytes and is higher in fat tissues of fat individuals than that in lean individuals across different species.

Compared to several existing papers that have studied sheep breeds from limited geographical areas and reported quite different candidates associated with fat tail phenotype [6–11], this study analyzed the largest cohort of samples until so far, to the best of our knowledge, originating from around the world. This practice avoids bias arising from geographic isolation or population structure and thus represents a more unambiguous and powerful strategy. Actually, our replication analysis of top SNPs proposed by previous studies in our large cohort reveals that most of these SNPs show genetic differentiation only between some regional sheep populations including SNPs corresponding to *BMP2*, which have been reported by several independent studies [7, 8, 11]. For instance, three SNPs, OAR13_51852034.1, ORA13_51886803.1 and s27419.1, at the *BMP2* locus, exhibited relatively higher DAF in fat-tailed sheep than that in thin-tailed (Additional file 10**: Fig. S4**). Unfortunately, the genetic differentiation level of the former two SNPs is below the genome-wide threshold in one (MEF vs SAT) and two group-pair comparison(s) (MEF vs SAT, MEF vs EUT), respectively (Additional file 2-4**: Table S2–4**). A possible reason for this is the low abundance of derived allele in some fat-tailed sheep populations, such as Afshari, LocalAwassi and EthiopianMenz (Additional file 10**: Fig. S4**). Therefore, *BMP2* has lower priority than the *PDGFD* locus as the candidate for fat-tailed phenotype. Furthermore, a recent study based on whole-genome re-sequencing data also found that *PDGFD* is the most highly differentiated loci between fat-tail sheep populations from China as well as Middle East, and thin-tailed Tibetan sheep, followed by *BMP2* as the second top highly divergent gene [8]. This report provided further support to our finding that *PDGFD* is the predominant candidate for fat tail in sheep, although our study suffers limitation that only genes covered by the Ovine 50 K Beadchip were investigated.

Adipose tissue development is associated with specification and differentiation of precursor cells (preadipocytes) to mature adipocytes as well as expansion of adipocyte size [14]. This finely orchestrated process involves changes in expression of a series of molecular regulators which remain to be discovered. Our morphological and histological analysis suggests that tail tissues undergo fat deposition from E60/E70 to E80/E90 in fat-tailed sheep during embryonic development (Fig. 3) and thus provides ideal materials for studying the molecular basis of adipogeneis. Gene expression analysis suggest that *PDGFD* expression is negatively associated with adipocyte accumulation (Fig. 4a-d). Consistently, re-analysis of data generated from previous studies in human and mouse reveals that *PDGFD* expression is enriched in preadipocytes and is decreased during the adipogenesis from precursor cells (Fig. 4e-f) [15, 16]. These observations strongly suggest that *PDGFD* plays a critical role in the initiation and/or commitment of preadipocytes and this function is conserved and universal across species. Despite a continuous decline of expression during development, we found that *PDGFD* sustains higher expression in tail tissues of fat-tailed sheep than that of thin-tailed sheep from embryonic (E70) to adult stage (Fig. 4h-i). Supporting this, a previous study reported that the expression of *PDGFD* is significantly upregulated in tail adipose tissue from fat-tailed Hulun Buir sheep as compared to thin-tailed Tibetan sheep [17]. Furthermore, another study showed that *PDGFD* is differentially expressed between tail adipose tissues from female Hulun Buir sheep with different levels of tail fat accumulation [18]. The higher expression of *PDGFD* could contribute to fat deposition by promoting the formation or proliferation of mature adipocytes, or altering lipid metabolism such as lipid synthesis, which needs to be further determined. More interestingly, this expression pattern holds true in mouse and human, with obese individuals exhibiting higher level of *PDGFD* expression in adipose tissues (Fig. 4j-k) [19, 20]. Together with a recent study reporting that *PDGFD* expression is associated with adipocyte number in human white adipose tissue [21], we propose that *PDGFD* is a novel regulator of adipogenesis and adipocyte hemostasis. Further gain/loss-of-function assays are necessary to confirm this potential function.

We discovered a list of mutations that are located within the first intron of *PDGFD* and narrowed down the candidate casual mutations to 13 SNPs which display high frequency in fat-tail sheep while low abundance in thin-tailed sheep (Fig. 2**and** Additional file 9**: Table S6**). Since intronic mutations mainly affect the transcriptional efficiency of genes by creating or disrupting binding sites for transcriptional factors [22, 23], it is likely that the elevated expression of *PDGFD* in fat-tailed sheep is attributed to some/one of these mutations. Future mutagenesis studies are warranted to determine the effect of mutated allele of these candidate SNPs identified in fat-tailed sheep on *PDGFD* expression to pinpoint the true casual mutation(s).

## Conclusions

In conclusion, we have demonstrated that *PDGFD* gene is the predominant factor underlying the fat tail phenotype in sheep and is a potentially novel regulator for adiopogenesis and maintaining hemostasis of mature adipocytes. Mutations occurring within the first intron of *PDGFD* is likely associated with *PDGFD* transcriptional activity and fat deposition. These findings are important for understanding the selection of the fat tail in sheep and useful in genome-based animal improvement, as well as obesity-related diseases in human.

## Methods

### Ethics statement

This study followed the recommendations of the “Regulations for the Management of Affairs Concerning Experimental Animals” (Ministry of Science and Technology, China, revised in June 2004). The study was approved by the ethics committees of all the participating institutes (Institute of Animal Science, Chinese Academy of Agricultural Sciences; Ningxia Academy of Agriculture and Forestry Sciences; Research Institute of Animal Science, Tibet Academy of Agricultural and Animal Husbandry Sciences National Animal Science Institute, Nepal Agricultural Research Council).

### Samples and genotyping

Genome-wide SNP data of 968 individual sheep from five South Asian thin-tailed sheep breeds (i.e., Tibetan, Changthangi, Deccani, IndianGarole and Garut sheep), six European thin-tailed sheep breeds (i.e., Churra, Leccese, Comisana, Altamurana, MacarthurMerino and MilkLacaune sheep), three American thin-tailed sheep breeds (i.e., BarbadosBlackBelly, MoradaNova and SantaInes sheep), six Middle East fat-tailed sheep breeds (i.e., Afshari, LocalAwassi, Karakas, Norduz, Moghani and CyprusFatTail sheep), four African fat-tailed sheep breeds (i.e., EthiopianMenz, NamaquaAfrikaner, RedMaasai and RonderibAfrikaner) and eight Mouflon sheep individuals (wild sheep) were downloaded from Ovine HapMap project (http://www.sheephapmap.org/hapmap.php) [24]. SNP data of four Chinese fat-tailed sheep breeds (i.e., Hu, Tong, Large tail Han and Lop sheep) were obtained from a previous study [9]. SNP data of four Nepalese thin-tailed sheep breeds (i.e., Bhyanglung, Baruwal, Lampuchhre and Kage sheep) were generated in our lab [25]. Tail type for these breeds was determined by either direct observation or description of previous literature. The detailed information of these sheep breeds was provided in Additional file 1**: Table S1**. All the SNP data were generated using the Illumina Ovine 50 K Beadchip and were thus readily merged together. The final dataset included 968 sheep individuals and 47,415 common autosomal SNPs (based on genome Oar_v3.1).

### Determination of ancestral allele and data quality control

Ancestral allele information for a subset of 33,059 SNPs were obtained from Ovine HapMap [24]. For the remaining SNPs, the ancestral allele was deduced according to the major allele in Mouflon sheep. Finally, a total of 46,540 SNPs with available ancestral allele information were kept for next analysis. PLINK v2.05 [26] was applied for further SNP data quality control. SNPs were removed if any of the following conditions were met: 1) with call rate ≤ 90%; 2) with minor allele frequency (MAF) ≤0.05. Individuals with an average call rate below 90% were discarded. To ensure independence among the studied sheep individuals, cryptic relatedness among individuals within each breed were identified using pair-wise Identity-By-Descent (IBD) metric (referred to as PI-HAT in PLINK). One individual from a pair of sheep individuals was removed from the following analyses if their PI-HAT value was over 0.3.

### Phylogenetic analysis

A pruned set of 32,450 SNPs were used to investigate the genetic relationship among these sheep breeds from different geographic locations. PCA was performed with the GCTA software [27] and the individuals outside of their expected population clusters were excluded from further analysis. The neighbor-joining tree was constructed using PHYLIP 3.68 (http://evolution.genetics.washington.edu/phylip.html) on the basis of allele frequency data. After PCA analysis, a total of 45,337 SNPs for 828 individuals from 30 diverse sheep breeds were retained for downstream analyses (Additional file 1**: Table S1**).

### Genomic screen for positively selected genes in fat-tailed sheep

Three previous studies compared the genomic variations between Middle East/European thin-tailed versus (v.s.) Middle East fat-tailed sheep [10], European thin-tailed v.s. Middle East fat-tailed sheep [11], and Chinese thin- versus fat-tailed sheep [9], respectively. Therefore, we used sheep breeds from South Asia, Middle East and Europe to identify the positively selected genes in fat-tailed sheep. Three group-pair comparisons between thin- and fat-tailed sheep were considered, including MEF v.s. SAT, MEF v.s. EUT, and CHF v.s. SAT.

Two statistics, including the *F*_*ST*_ and ΔDAF were applied to evaluate the genetic differentiation of each SNP between thin- and fat-tailed sheep populations. The *F*_*ST*_ analysis was conducted using Genepop 4.3 software [28]. ΔDAF was calculated as the derived allele frequency in the fat-tailed sheep minus the DAF in thin-tailed sheep (DAF_Fat-tailed sheep_ – DAF_Thin-tailed sheep_) using R version 3.3.3 (https://www.r-project.org/). For each group-pair comparison, *F*_*ST*_ and ΔDAF at each SNP marker was calculated between each thin-tailed breed against each fat-tailed breed and averaged across breed-pairs to produce an overall value for each SNP. The top 1% SNPs with large overall *F*_*ST*_ or ΔDAF value were considered as the significant SNPs in each test and the significant SNPs overlapped in both tests were considered as positively selected SNPs in fat-tailed sheep. Finally, positively selected SNPs that were identified in all three group-pair comparisons were considered as the loci under positive selection in fat-tailed sheep and genes within 150 kb of these loci were retrieved from Ensembl BioMart database (http://useast.ensembl.org/biomart/martview/625a397ecca62a421509935f099cdaa7). Because the high average value of *F*_*ST*_ or ΔDAF may have resulted from the extremely large values in several specific breed-pairs due to population structure, we additionally examined the DAF of the candidate SNPs in three thin-tailed sheep breeds from Americas and three fat-tailed sheep breeds from Africa and further filtered the promising candidate list by removing SNPs with DAF > 0.5 in more than five thin-tailed sheep breeds or DAF < 0.5 in more than four fat-tailed sheep breeds (> 30% of total thin- or fat-tailed sheep breeds).

### Analysis of sequence variations within top genes

Genomic variations stored in Variant Call Format (VCF) file for 16 thin-tailed sheep individuals and 13 fat-tailed sheep individuals from 17 different breeds from around the world were downloaded from NextGen of Ensembl Projects (http://projects.ensembl.org/nextgen/) (Additional file 7**: Table S5**). The VCF file was converted to PLINK PED file using VCFtools [29]. PLINK [26] was used to perform the SNP quality control (removing SNPs with call rate ≤ 90% or MAF ≤0.05) and to calculate the allele frequency of each SNP in thin- and fat-tailed sheep group. The absolute ΔAF) of each SNP between the fat- and thin-tailed sheep group were calculated using R.

### Validation of top SNPs in expanded samples using Sequenom MassARRAY

Ear tissues of 200 Tibetan sheep (Thin-tailed sheep) 184 Tan sheep (fat-tailed sheep) were collected from farms operated by the Research Institute of Animal Science, Tibet Academy of Agricultural and Animal Husbandry Sciences, and Ningxia Yanchi Tan sheep breeding farm, respectively. Animals were released after collecting the samples. Genomic DNA was isolated and was quantified using a Nanodrop 2000 (Thermo Fisher Scientific, DE). A total of 13 intronic SNPs of *PDGFD* gene with the highest ΔAF value (> 0.8) and derived allele frequency larger than 0.8 in fat-tailed sheep were selected for further validation in this expanded cohort. The genotyping of these 13 SNPs was carried out with Sequenom MassArray system. Briefly, primers for PCR and for locus-specific single-base extension were designed with MassArray Assay Design 4.0. The PCR products were applied for locus-specific single-base extension reactions. The final products were desalted and transferred to a matrix chip. The resultant mass spectrograms and genotypes were analyzed with MassArray Typer software.

### Sections, Hematoxylin and eosin (HE) staining, oil red O staining

A total of 16 pregnant tan sheep individuals (fat-tailed sheep) subjected to artificial insemination were randomly chosen at farm of Institute of Animal Science, Ningxia Academy of Agricultural and Forestry Sciences in China. At each of the expected embryonic stages including embryonic day 60 (E60), E70, E80 and E90, four animals were euthanized by captive bolt stunning followed by exsanguination and embryonic tail tissues were collected and subjected to histology analysis, RNA-seq analysis or qRT-PCR. For Histology analysis, tissues were fixed with 4% paraformaldehyde over-night at 4 °C and then embedded in paraffin. Sections were cut at 8-um thickness. For HE staining, sections were deparaffinized with two changes of Xylene (10 min each) and were rehydrated with 100, 95 and 80% ethanol rinses (5 min each). After a brief wash in distilled water, sections were stained in hematoxylin solution for 5 min, washed in running tap water for 5 min, differentiated in 1% acid alcohol for 30 s and washed again with running tap water for 1 min. Following bluing in 0.2% ammonia water for 30 s and a wash in running tap water for 5 min, sections were rinsed in 95% alcohol and stained in eosin-phloxine solution for 30 s. Sections were then dehydrated with a series of rinses in 80% alcohol (5 s), 95% alcohol (twice, 10 s each), 100% alcohol (twice, 5 min each), and then mounted. For Oil Red O staining, 0.5 g Oil Red O powder (Sigma) was evenly dissolved in 100 mL of isopropanol to prepare stock solution, which was then diluted with distilled water at the ratio of 3:2 and filtrated to make working solution. The sections were rinsed with 60% isopropanol and then stained with Oil Red O working solution for 15 min. Sections were then mounted and imaged for evaluation of droplet formation.

### RNA-sequencing and bioinformatics analysis

Tail tissues from Tan sheep at three different developmental stages as described above including E60, E70, E80 were used for RNA-seq analysis. Tail tissues were flash frozen in liquid nitrogen and stored at − 80 °C for subsequent RNA extraction. Total RNA was extracted by RNeasy Lipid Tissue Mini Kit (Qiagen) and the RNA-seq library was constructed with Dynabeads mRNA DIRECT Kit (invitrogen) for whole transcriptome RNA-seq analysis. Pair-end RNA-sequencing was performed on a X-ten system (Illumina) in 150-bp length.

After removing adaptor sequence and low-quality reads, pass-filtered reads were then mapped to UCSC sheep reference genome Oar_v4.0 using Tophat 2.1.1 [30]. The genes annotated in RefSeq were quantified with Cufflinks [31]. FPKM (Fragments Per Kilobase of exon per Million fragments mapped) were calculated from raw counts and used to quantify relative gene expression for each sample.

### Quantitative reverse transcription-PCR (qRT-PCR) analysis

Tail tissues of Tan sheep (fat-tailed) at different developmental stages including E60, E70, E80, E90 described above were used for qRT-PCR analysis of *PDGFD* expression during development. Four additional tail samples from each of Suffolk sheep (thin-tailed sheep) at E70 and Tan sheep at adult stage (about 2 years old, female) were collected from farm operated by the Institute of Animal Science, Ningxia Academy of Agricultural and Forestry Sciences in China. Tail samples from four Bhyanglung sheep individuals at adult stage (thin-tailed sheep, about 2 years old, female) were collected from farm of National Animal Science Institute, Nepal Agricultural Research Council in Nepal. For embryonic sample collection, animals were euthanized by captive bolt stunning followed by exsanguination and embryonic tail tissues were harvested. For adult sample collection, animals were released right after harvesting samples. Total RNA from tail tissues were isolated with RNeasy Lipid Tissue Mini Kit (Qiagen). 0.8 μg of total RNA was utilized as template for RT with random hexamer primers using PrimeScript RT reagent Kit (Takara). qRT-PCR was performed with respective gene-specific primers (*PDGFD:* Forward: GGGAGTCAGTCACAAGCTCT, Reverse: AGTGGGGTCCGTTACTGATG; *ACTB*: Forward: TCTGGCACCACACCTTCTAC; Reverse: TCTTCTCACGGTTGGCCTTG). All samples were amplified in triplicate and all experiments were repeated at least 3 independent times. Relative gene expression was converted using the 2^-∆∆CT^ method against the internal control house-keeping gene ACTB where ∆∆CT = (CT_experimental gene_ - CT_experimental ACTB_) - (CT_control gene_ - CT_control ACTB_). The relative gene expression in control group was set to 1. Student t test was performed to determine significance.

### *PDGFD* expression in adipose tissues/cells in mouse and human

Micro-array data of different cell types isolated from human white adipose tissues by fluorescence-activated cell sorting (FACS) including CD45−/CD34+/CD31- (progenitors), CD45+/CD14+/CD206+ (total macrophages), CD45+/CD14 + CD206+/CD11c + (M1 macrophages), CD45+/CD14+/CD206+/CD11c- (M2 macrophages), CD45+/CD3+ (Total T cells), CD45+/CD3+/CD4+/CD8- (Th T-cells), CD45+/CD3+/CD4−/CD8+ (Tc T-cells) (GSE100795) [16] were obtained from GEO database and re-analyzed with online build-in tool GEO2R. Raw gene count matrix generated from RNA-seq for mouse 3 T3-L1 preadiopocytes and differentiating cells at 24 h after induction by DHA differentiation cocktail was obtained from GEO database (GSE118471) [15]. FPKM was calculated for each sample and used for quantification of gene expression level. Micro-array data of inguinal fat tissues from mice exhibiting high or low weight gain after 4 weeks on a high saturated fat diet (GSE4692) [20], adipocytes from non-diabetic lean and non-diabetic obese Pima Indian subjects (GSE2508) [19] were obtained from GEO database and analyzed with online build-in tool GEO2R.

## Supplementary Information

**Additional file 1 Table S1**. Information of samples used in this study.

**Additional file 2 Table S2**. Positively selected SNPs identified in comparison of Middle East fat-tailed sheep vs South Asian thin-tailed sheep.

**Additional file 3 Table S3**. Positively selected SNPs identified in comparison of Middle East fat-tailed sheep vs European thin-tailed sheep.

**Additional file 4 Table S4**. Positively selected SNPs identified in comparison of Chinese fat-tailed sheep vs South Asian thin-tailed sheep.

**Additional file 5 Figure S1**. The distribution of derived allele frequency (DAF) of the 16 candidate SNPs in each breed. SNPs were sorted according to the average value of FST and ΔDAF among the three comparisons indicated in Fig. 1b from high to low. SNPs labeled in red represented promising candidate SNPs for fat tail in sheep and the remaining eight SNPs were excluded from the promising candidate list because they failed to pass the filter criterial described in Methods.

**Additional file 6 Figure S2**. Phylogenetic analysis of the studied sheep breeds. (A) Principle Component Analysis (PCA) results. The left panel shows the PCA plot generated based on genome wide SNPs. The right panel shows the PCA plot generated based on the 8 candidate SNPs. (B) Phylogenetic tree results. The left panel shows the phylogenetic tree constructed according to genome wide SNPs. The right panel shows the phylogenetic tree constructed according to the 8 candidate SNPs.

**Additional file 7 Table S5**. Information of samples used for genome sequence variation analysis.

**Additional file 8 Figure S3**. The expression level across different tissues of *PDGFD* gene obtained from FANTOM5 dataset from The Human Protein Atlas database.

**Additional file 9 Table S6**. Highly differentiated SNPs (ΔAF > 0.60) within the putative targeted region of positive selection in *PDGFD* gene (Chr15:3,854,063-3,860,894 bp).

**Additional file 10 Figure S4**. Replication of the top candidate SNPs proposed by three previous studies in all sheep breeds used in this study. **(A)** Derived allele frequency (DAF) of the top candidate SNPs previously proposed. **(B)** The top panel of “**A**” indicates the the derived allele frequency (DAF) of 10 top SNPs on chromosome 5 and 7 proposed in Table 3 in study of Moradi et al.. The s553221.1 locus on chromosome 5 in their study was not included because it did not pass the quality control in this study. **(B)** The middle panel of “**A**” shows the DAF of four SNPs corresponding to *BMP2* and two SNPs corresponding to *VRTN* gene proposed in Table 2 in study of Moioli et al.. The s73063.1 locus annotated to *VRTN* was not was not included because it did not pass the quality control in this study. **(C)** The bottom panel of “**A**” represents the DAF of SNPs annotated to the key genes proposed in Table 1 and Table 2 in study of Yuan et al. The OAR4_73050615.1 locus is from Table 1 and other loci are from Table 2 in their study.

**Additional file 11 Figure S5.** Expression of the top candidate genes in tail tissues of fat-tailed sheep during embryonic development revealed by RNA-seq**.** E60: embryonic day 60. E70: embryonic day 70. E80: embryonic day 80.

## Data Availability

The SNP Beadchip datasets supporting the conclusions of this article are included within the article and its additional files. The RNA-seq data of sheep tail tissues were deposited in Sequence Read Archive (SRA) under accession number PRJNA665351.

## Abbreviations

SNP: Single nucleotide polymorphism
MAF: Minor allele frequency
IBD: Identify-By-Descent
PI-HAT: Proportion IBD
FST: Genetic Fixation Index
ΔDAF: Difference of derived allele frequency
MEF: Middle East fat-tailed sheep
SAT: South Asian thin-tailed sheep
EUT: European thin-tailed sheep
CHF: Chinese fat-tailed sheep
VCF: Variant Call Format
HE: Hematoxylin and Eosin
E60, E70, E80, E90: Embryonic day 60, 70, 80, 90
FPKM: Fragments Per Kilobase of exon per Million fragments mapped
qRT-PCR: Quantitative reverse transcription-PCR
FACS: Fluorescence-activated cell sorting
