# Supplementary material for: Genomic analysis of worldwide sheep breeds reveals PDGFD as a major target of fat-tail selection in sheep
Source: BMC Genomics. 2020 Nov 17;21:800. doi: 10.1186/s12864-020-07210-9 (PMC7670677; doi:10.1186/s12864-020-07210-9)
Supplement: Supplementary file 1 — Additional file 1 Table S1. Information of samples used in this study. [file 12864_2020_7210_MOESM1_ESM.doc]

Table S1. Information of samples used in this study

| **Population** | **Number** | **Number after relatedness analysis** | **Number after PCA analysis** | **Origin** | **Country** | **Tail type** |
| --- | --- | --- | --- | --- | --- | --- |
| Tibetan | 37 | 35 | 35 | South Asia | China | Thin |
| Changthangi | 29 | 25 | 20 | South Asia | India | Thin |
| Bhyanglung | 24 | 19 | 19 | South Asia | Nepal | Thin |
| Baruwal | 24 | 10 | 10 | South Asia | Nepal | Thin |
| Lampuchhre | 24 | 20 | 20 | South Asia | Nepal | Thin |
| Kage | 24 | 12 | 11 | South Asia | Nepal | Thin |
| Deccani | 24 | 23 | 20 | South Asia | India | Thin |
| IndianGarole | 26 | 21 | 21 | South Asia | India | Thin |
| Garut | 22 | 21 | 18 | South Asia | Indonesia | Thin |
| Churra | 120 | 101 | 101 | Europe | Spain | Thin |
| Leccese | 24 | 20 | 20 | Europe | Italy | Thin |
| Comisana | 24 | 24 | 24 | Europe | Italy | Thin |
| Altamurana | 24 | 23 | 23 | Europe | Italy | Thin |
| MacarthurMerino | 10 | 5 | 0 | Europe | Unknown | Thin |
| MilkLacaune | 103 | 100 | 100 | Europe | France | Thin |
| BarbadosBlackBelly | 24 | 21 | 21 | Americas | U.S. | Thin |
| MoradaNova | 22 | 19 | 19 | Americas | Brazil | Thin |
| SantaInes | 47 | 43 | 43 | Americas | Brazil | Thin |
| Afshari | 37 | 35 | 34 | Middle East | Iran | Fat |
| LocalAwassi | 24 | 23 | 23 | Middle East | Unknown | Fat |
| Karakas | 18 | 15 | 15 | Middle East | Turkey | Fat |
| Norduz | 20 | 19 | 19 | Middle East | Turkey | Fat |
| Moghani | 34 | 34 | 34 | Middle East | Iran | Fat |
| CyprusFatTail | 30 | 25 | 25 | Middle East | Turkey | Fat |
| HUS | 12 | 12 | 12 | East Asia | China | Fat |
| TON | 15 | 15 | 15 | East Asia | China | Fat |
| LTH | 15 | 14 | 14 | East Asia | China | Fat |
| LOP | 15 | 13 | 23 | East Asia | China | Fat |
| EthiopianMenz | 34 | 34 | 34 | Africa | Ethiopia | Fat |
| NamaquaAfrikaner | 12 | 4 | 0 | Africa | South Africa | Fat |
| RedMaasai | 45 | 35 | 34 | Africa | Kenya | Fat |
| RonderibAfrikaner | 17 | 13 | 13 | Africa | Unknown | Fat |
| Mouflon | 8 | 8 | 8 | Unknown | Unknown | Thin |
