# Supplementary material for: Genomic analysis of worldwide sheep breeds reveals PDGFD as a major target of fat-tail selection in sheep
Source: BMC Genomics. 2020 Nov 17;21:800. doi: 10.1186/s12864-020-07210-9 (PMC7670677; doi:10.1186/s12864-020-07210-9)
Supplement: Supplementary file 2 — Additional file 2 Table S2. Positively selected SNPs identified in comparison of Middle East fat-tailed sheep vs South Asian thin-tailed sheep. [file 12864_2020_7210_MOESM2_ESM.doc]

Table S2. Positively selected SNPs identified in comparison of Middle East fat-tailed sheep vs South Asian thin-tailed sheep

| **CHR** | **SNP** | **Position** | **ancestralAllele** | **derivedAllele** | **ΔDAF** | **FST** |
| --- | --- | --- | --- | --- | --- | --- |
| 1 | s72240.1 | 94802323 | A | G | 0.378 | 0.282 |
| 1 | OAR1_100874172.1 | 94940617 | A | G | 0.345 | 0.314 |
| 1 | OAR1_100921673.1 | 94993712 | G | A | 0.556 | 0.489 |
| 1 | s54125.1 | 96084447 | A | T | 0.397 | 0.295 |
| 1 | OAR1_112929344.1 | 105175111 | A | G | 0.422 | 0.318 |
| 1 | OAR1_131165545.1 | 121009077 | G | A | 0.362 | 0.296 |
| 1 | OAR1_134035072.1 | 123661951 | A | G | 0.359 | 0.298 |
| 1 | s32216.1 | 128983286 | G | A | 0.437 | 0.399 |
| 1 | OAR1_142588286.1 | 131702493 | A | C | 0.365 | 0.288 |
| 1 | OAR1_147643165_X.1 | 136675253 | G | A | 0.418 | 0.321 |
| 1 | OAR1_165683429.1 | 153939997 | A | G | 0.419 | 0.329 |
| 1 | OAR1_172690647.1 | 160114886 | G | A | 0.375 | 0.280 |
| 1 | OAR1_206552578.1 | 191356092 | A | G | 0.404 | 0.331 |
| 1 | OAR1_208070059.1 | 192689940 | G | A | 0.347 | 0.291 |
| 1 | s46936.1 | 239250765 | A | G | 0.364 | 0.300 |
| 1 | OAR1_276539228.1 | 255958836 | A | G | 0.391 | 0.293 |
| 1 | OAR1_294410597.1 | 271906062 | A | G | 0.410 | 0.341 |
| 1 | s43083.1 | 272672766 | C | A | 0.407 | 0.319 |
| 2 | OAR2_13939688.1 | 14523912 | G | A | 0.376 | 0.324 |
| 2 | OAR2_14510169.1 | 15061596 | G | A | 0.563 | 0.511 |
| 2 | s48303.1 | 15316224 | G | A | 0.458 | 0.347 |
| 2 | OAR2_14833429.1 | 15388156 | G | A | 0.380 | 0.307 |
| 2 | OAR2_31404234_X.1 | 30485387 | G | A | 0.562 | 0.480 |
| 2 | OAR2_31412377.1 | 30492514 | G | A | 0.543 | 0.465 |
| 2 | OAR2_32694090.1 | 31519390 | A | G | 0.410 | 0.321 |
| 2 | OAR2_48310588_X.1 | 45500785 | A | G | 0.590 | 0.531 |
| 2 | s73578.1 | 58258740 | A | G | 0.412 | 0.324 |
| 2 | s00950.1 | 81689824 | G | A | 0.418 | 0.315 |
| 2 | DU314213_585.1 | 86465561 | G | A | 0.364 | 0.294 |
| 2 | OAR2_113952627.1 | 105984105 | A | G | 0.397 | 0.307 |
| 2 | OAR2_114078125.1 | 106109303 | A | G | 0.396 | 0.294 |
| 2 | OAR2_118934331.1 | 110699239 | A | G | 0.387 | 0.325 |
| 2 | OAR2_156740619.1 | 147804448 | A | G | 0.577 | 0.493 |
| 2 | s60772.1 | 148013217 | G | A | 0.552 | 0.468 |
| 2 | OAR2_172701586.1 | 163070840 | G | A | 0.563 | 0.504 |
| 2 | OAR2_192983948.1 | 182124730 | G | A | 0.423 | 0.390 |
| 2 | OAR2_193444466_X.1 | 182607165 | G | A | 0.680 | 0.649 |
| 2 | OAR2_194090177.1 | 183164212 | A | G | 0.389 | 0.329 |
| 2 | s23870.1 | 183999143 | A | G | 0.451 | 0.357 |
| 2 | OAR2_201079081_X.1 | 189708780 | C | A | 0.403 | 0.308 |
| 2 | s14470.1 | 192231314 | A | G | 0.565 | 0.497 |
| 2 | OAR2_204059664.1 | 192530617 | G | A | 0.417 | 0.302 |
| 2 | OAR2_236955619.1 | 224283993 | G | A | 0.445 | 0.334 |
| 2 | s62767.1 | 232469995 | G | A | 0.337 | 0.280 |
| 2 | s39367.1 | 245683288 | A | G | 0.393 | 0.310 |
| 3 | s45829.1 | 26620705 | G | A | 0.407 | 0.301 |
| 3 | s52457.1 | 26663352 | A | G | 0.466 | 0.380 |
| 3 | s48427.1 | 26936637 | G | A | 0.412 | 0.286 |
| 3 | OAR3_43027143.1 | 40285089 | A | C | 0.416 | 0.294 |
| 3 | OAR3_45819663.1 | 42791937 | A | G | 0.407 | 0.295 |
| 3 | OAR3_96170847.1 | 90549311 | C | A | 0.498 | 0.443 |
| 3 | s74187.1 | 90919722 | G | A | 0.381 | 0.291 |
| 3 | s66125.1 | 93671110 | G | A | 0.406 | 0.336 |
| 3 | OAR3_99622529.1 | 93811641 | G | A | 0.444 | 0.331 |
| 3 | OAR3_113183544.1 | 106356555 | A | C | 0.377 | 0.311 |
| 3 | OAR3_114169629.1 | 107281051 | C | A | 0.384 | 0.310 |
| 3 | OAR3_114225433.1 | 107334356 | A | G | 0.587 | 0.534 |
| 3 | OAR3_126893362.1 | 118982570 | A | G | 0.461 | 0.411 |
| 3 | OAR3_138290871.1 | 129648614 | G | A | 0.464 | 0.401 |
| 3 | OAR3_138331159.1 | 129685397 | G | A | 0.380 | 0.303 |
| 3 | OAR3_141586525.1 | 132478420 | G | A | 0.742 | 0.700 |
| 3 | OAR3_141681710.1 | 132527834 | A | G | 0.487 | 0.399 |
| 3 | OAR3_141703798.1 | 132549121 | G | A | 0.553 | 0.475 |
| 3 | s50426.1 | 133886147 | G | A | 0.517 | 0.416 |
| 3 | s31828.1 | 134177731 | G | A | 0.343 | 0.308 |
| 3 | OAR3_165050963.1 | 154252449 | G | A | 0.542 | 0.462 |
| 3 | OAR3_172957287.1 | 161755779 | G | C | 0.461 | 0.364 |
| 3 | OAR3_183605277.1 | 171004273 | G | A | 0.423 | 0.374 |
| 3 | s08427.1 | 179895839 | A | G | 0.419 | 0.326 |
| 3 | OAR3_200125923.1 | 185881136 | A | G | 0.678 | 0.637 |
| 3 | OAR3_200805613.1 | 186593583 | A | G | 0.507 | 0.414 |
| 3 | OAR3_200820005_X.1 | 186613455 | G | A | 0.533 | 0.448 |
| 3 | OAR3_200934301.1 | 186702791 | A | G | 0.494 | 0.426 |
| 3 | OAR3_208496550.1 | 193780632 | G | A | 0.382 | 0.291 |
| 3 | s24239.1 | 218572118 | G | A | 0.437 | 0.333 |
| 4 | s60665.1 | 10059949 | G | A | 0.425 | 0.319 |
| 4 | OAR4_51625352.1 | 48804045 | G | A | 0.362 | 0.312 |
| 4 | s33255.1 | 65021659 | A | G | 0.437 | 0.335 |
| 4 | OAR4_69915105.1 | 65950573 | A | G | 0.403 | 0.303 |
| 4 | OAR4_72150069.1 | 68164406 | A | C | 0.410 | 0.362 |
| 4 | OAR4_72440076.1 | 68468908 | G | A | 0.394 | 0.339 |
| 4 | s25289.1 | 68802676 | A | G | 0.408 | 0.317 |
| 4 | OAR4_84273713.1 | 79447900 | A | C | 0.391 | 0.296 |
| 4 | OAR4_99003884.1 | 93383949 | G | A | 0.395 | 0.303 |
| 5 | OAR5_17894545.1 | 15522700 | G | A | 0.390 | 0.345 |
| 5 | OAR5_20120209.1 | 17557416 | A | C | 0.395 | 0.323 |
| 5 | s33925.1 | 17890738 | C | A | 0.388 | 0.390 |
| 5 | OAR5_47263230.1 | 43236671 | G | A | 0.420 | 0.305 |
| 5 | OAR5_73558894.1 | 66883998 | G | A | 0.362 | 0.302 |
| 6 | OAR6_37483582.1 | 33493183 | A | C | 0.367 | 0.287 |
| 6 | OAR6_40243862.1 | 36040916 | A | G | 0.369 | 0.350 |
| 6 | OAR6_40370293.1 | 36155169 | A | G | 0.653 | 0.595 |
| 6 | OAR6_50201736.1 | 45276382 | G | A | 0.481 | 0.371 |
| 6 | OAR6_64965984.1 | 58974752 | A | C | 0.327 | 0.287 |
| 6 | OAR6_74570690.1 | 68044077 | G | A | 0.400 | 0.320 |
| 6 | OAR6_92321965.1 | 84350628 | G | A | 0.506 | 0.429 |
| 6 | OAR6_103468877.1 | 94394364 | A | G | 0.392 | 0.323 |
| 6 | OAR6_120771496.1 | 106319428 | A | C | 0.388 | 0.310 |
| 6 | OAR6_125373929.1 | 110431665 | A | G | 0.384 | 0.298 |
| 7 | OAR7_35146905.1 | 31225687 | G | A | 0.402 | 0.310 |
| 7 | OAR7_36740905.1 | 32557019 | G | A | 0.443 | 0.368 |
| 7 | s07104.1 | 63406397 | G | A | 0.395 | 0.304 |
| 7 | OAR7_97159644.1 | 89286934 | G | A | 0.522 | 0.438 |
| 8 | OAR8_12517372.1 | 11173594 | G | A | 0.433 | 0.334 |
| 8 | OAR8_34033040.1 | 31255521 | A | C | 0.625 | 0.562 |
| 8 | OAR8_34133754.1 | 31339677 | G | A | 0.582 | 0.503 |
| 8 | OAR8_55825703.1 | 51981196 | A | C | 0.373 | 0.343 |
| 8 | OAR8_56910466.1 | 53042000 | G | A | 0.432 | 0.346 |
| 8 | OAR8_58028748.1 | 54142002 | A | G | 0.368 | 0.286 |
| 8 | OAR8_62425494.1 | 58088530 | G | A | 0.436 | 0.325 |
| 9 | OAR9_31854883.1 | 30459575 | C | A | 0.338 | 0.280 |
| 9 | OAR9_38221892.1 | 36285902 | A | G | 0.472 | 0.374 |
| 9 | OAR9_70187264.1 | 66214217 | G | A | 0.405 | 0.299 |
| 9 | OAR9_86134472.1 | 81458809 | G | A | 0.450 | 0.330 |
| 9 | OAR9_99601991.1 | 93576726 | G | A | 0.425 | 0.387 |
| 10 | OAR10_18038138.1 | 18930071 | A | G | 0.497 | 0.397 |
| 10 | OAR10_19307844.1 | 19979392 | A | G | 0.356 | 0.284 |
| 10 | s45528.1 | 20551435 | G | A | 0.421 | 0.359 |
| 10 | OAR10_19917915.1 | 20584964 | G | A | 0.424 | 0.365 |
| 10 | OAR10_28198432.1 | 28183710 | A | G | 0.358 | 0.284 |
| 10 | s32389.1 | 38727795 | A | G | 0.510 | 0.424 |
| 10 | OAR10_53308606.1 | 52272053 | A | G | 0.415 | 0.294 |
| 10 | OAR10_53515386.1 | 52459290 | A | G | 0.454 | 0.341 |
| 10 | s33301.1 | 84332248 | A | G | 0.370 | 0.282 |
| 11 | s50820.1 | 8911693 | G | A | 0.468 | 0.376 |
| 11 | OAR11_18701428.1 | 18325488 | G | A | 0.911 | 0.907 |
| 11 | OAR11_18815864.1 | 18433474 | G | A | 0.894 | 0.888 |
| 11 | OAR11_18823250.1 | 18440783 | A | G | 0.851 | 0.847 |
| 11 | s69909.1 | 18581219 | G | A | 0.396 | 0.315 |
| 11 | OAR11_19810690.1 | 19407955 | G | A | 0.423 | 0.333 |
| 11 | OAR11_20163009.1 | 19726125 | T | A | 0.404 | 0.299 |
| 11 | s41017.1 | 22814442 | A | G | 0.324 | 0.290 |
| 11 | OAR11_27752920.1 | 26512466 | G | A | 0.418 | 0.315 |
| 11 | OAR11_36045417.1 | 33722495 | G | A | 0.380 | 0.299 |
| 11 | OAR11_39358742.1 | 36917331 | G | A | 0.446 | 0.372 |
| 11 | OAR11_39377571.1 | 36938798 | A | G | 0.448 | 0.376 |
| 11 | OAR11_39717067.1 | 37283862 | A | G | 0.331 | 0.325 |
| 11 | OAR11_47522525.1 | 44691892 | G | A | 0.473 | 0.384 |
| 11 | OAR11_52406539.1 | 49332243 | A | C | 0.411 | 0.329 |
| 12 | OAR12_40366545.1 | 36154610 | A | G | 0.408 | 0.292 |
| 12 | OAR12_43199598.1 | 38805757 | A | C | 0.483 | 0.426 |
| 12 | OAR12_43331187.1 | 38923821 | G | C | 0.372 | 0.348 |
| 12 | OAR12_62615870.1 | 56261324 | G | A | 0.375 | 0.280 |
| 12 | OAR12_72924306.1 | 66252130 | G | A | 0.577 | 0.525 |
| 12 | s36170.1 | 77590625 | A | C | 0.325 | 0.312 |
| 13 | s27965.1 | 7743780 | G | A | 0.422 | 0.311 |
| 13 | OAR13_51727898.1 | 48493120 | A | C | 0.414 | 0.323 |
| 13 | s40901.1 | 48553033 | A | G | 0.385 | 0.282 |
| 13 | OAR13_51817610.1 | 48585628 | C | A | 0.448 | 0.332 |
| 13 | s27419.1 | 48968332 | G | A | 0.461 | 0.401 |
| 13 | OAR13_60759835.1 | 55866953 | G | A | 0.402 | 0.325 |
| 13 | OAR13_60821868.1 | 55934178 | G | A | 0.379 | 0.301 |
| 13 | OAR13_62456006.1 | 57337743 | G | A | 0.415 | 0.344 |
| 13 | s74018.1 | 75451165 | G | A | 0.438 | 0.348 |
| 14 | s52780.1 | 13058130 | G | A | 0.419 | 0.302 |
| 14 | s66108.1 | 34390195 | G | A | 0.421 | 0.336 |
| 14 | OAR14_36887906.1 | 35453538 | A | G | 0.442 | 0.344 |
| 14 | s36271.1 | 35607975 | A | G | 0.344 | 0.281 |
| 14 | OAR14_51753220.1 | 49135580 | G | A | 0.384 | 0.297 |
| 14 | OAR14_63638742.1 | 58296635 | A | G | 0.414 | 0.339 |
| 15 | OAR15_2999185.1 | 3499482 | G | A | 0.451 | 0.409 |
| 15 | OAR15_3091174.1 | 3706790 | G | A | 0.761 | 0.732 |
| 15 | OAR15_3236575.1 | 3875564 | C | A | 0.346 | 0.308 |
| 15 | OAR15_37443121.1 | 35597025 | G | A | 0.410 | 0.284 |
| 15 | OAR15_37561366.1 | 35721980 | G | A | 0.376 | 0.284 |
| 15 | OAR15_65334817.1 | 59860515 | G | A | 0.415 | 0.360 |
| 15 | s15726.1 | 72924539 | G | A | 0.380 | 0.319 |
| 16 | OAR16_15458830.1 | 14215465 | A | G | 0.394 | 0.293 |
| 16 | OAR16_32336229.1 | 29759501 | G | A | 0.383 | 0.302 |
| 16 | OAR16_42159705.1 | 38851083 | A | G | 0.447 | 0.397 |
| 16 | OAR16_42321024.1 | 38952447 | A | G | 0.496 | 0.421 |
| 16 | s50768.1 | 57094224 | G | A | 0.392 | 0.319 |
| 17 | OAR17_7039737.1 | 6377686 | A | C | 0.447 | 0.343 |
| 17 | OAR17_20431552.1 | 18187471 | A | G | 0.401 | 0.306 |
| 17 | OAR17_31561091.1 | 28790576 | A | G | 0.348 | 0.286 |
| 17 | OAR17_36966397.1 | 33954083 | A | G | 0.558 | 0.504 |
| 17 | OAR17_58064576.1 | 53350131 | A | G | 0.390 | 0.356 |
| 17 | s09129.1 | 53470559 | A | G | 0.350 | 0.300 |
| 17 | s49475.1 | 53499969 | G | A | 0.355 | 0.309 |
| 17 | s11414.1 | 57552051 | A | G | 0.378 | 0.284 |
| 18 | OAR18_31974215.1 | 30706077 | A | G | 0.347 | 0.287 |
| 19 | s30230.1 | 3793724 | A | G | 0.360 | 0.323 |
| 19 | s38567.1 | 7169893 | G | A | 0.556 | 0.500 |
| 19 | OAR19_14430440.1 | 13866655 | A | G | 0.403 | 0.286 |
| 19 | OAR19_29776311.1 | 28134366 | G | A | 0.396 | 0.306 |
| 19 | OAR19_30535417.1 | 28943148 | A | G | 0.413 | 0.310 |
| 19 | s74549.1 | 29047903 | A | C | 0.447 | 0.406 |
| 19 | s63765.1 | 45498763 | G | A | 0.410 | 0.296 |
| 19 | s08944.1 | 55660459 | G | A | 0.412 | 0.337 |
| 19 | OAR19_60184770.1 | 56634864 | G | A | 0.443 | 0.359 |
| 20 | OAR20_5074340.1 | 5055888 | A | G | 0.379 | 0.282 |
| 20 | s11798.1 | 6907543 | A | G | 0.384 | 0.328 |
| 21 | OAR21_2948452.1 | 2268505 | G | A | 0.429 | 0.315 |
| 21 | s57643.1 | 2505963 | G | A | 0.387 | 0.294 |
| 21 | OAR21_3367762.1 | 2549106 | C | A | 0.364 | 0.309 |
| 21 | OAR21_12631355.1 | 11064132 | G | A | 0.374 | 0.320 |
| 21 | s33980.1 | 39638962 | A | G | 0.530 | 0.452 |
| 21 | s25535.1 | 39691463 | G | A | 0.416 | 0.384 |
| 21 | OAR21_43646542.1 | 39701145 | A | C | 0.444 | 0.378 |
| 22 | OAR22_34323192.1 | 29843623 | A | G | 0.411 | 0.358 |
| 22 | s19503.1 | 36806429 | G | A | 0.482 | 0.383 |
| 22 | s02448.1 | 40450104 | C | A | 0.466 | 0.376 |
| 22 | OAR22_45509727.1 | 40482146 | G | A | 0.430 | 0.348 |
| 22 | s47182.1 | 47229488 | A | G | 0.348 | 0.295 |
| 23 | OAR23_62745446.1 | 58943571 | A | C | 0.409 | 0.285 |
| 23 | s30046.1 | 61590863 | A | G | 0.356 | 0.296 |
| 24 | OAR24_29236630.1 | 26681408 | G | A | 0.423 | 0.388 |
| 24 | s02082.1 | 33413892 | G | A | 0.387 | 0.289 |
| 24 | s06024.1 | 34985988 | G | A | 0.392 | 0.305 |
| 24 | s08464.1 | 35991517 | G | A | 0.467 | 0.423 |
| 24 | s34514.1 | 36036418 | G | A | 0.433 | 0.361 |
| 25 | OAR25_38239942_X.1 | 36434142 | G | A | 0.404 | 0.286 |
| 26 | OAR26_33131883.1 | 28902195 | A | G | 0.393 | 0.286 |
