# Supplementary material for: Genomic analysis of worldwide sheep breeds reveals PDGFD as a major target of fat-tail selection in sheep
Source: BMC Genomics. 2020 Nov 17;21:800. doi: 10.1186/s12864-020-07210-9 (PMC7670677; doi:10.1186/s12864-020-07210-9)
Supplement: Supplementary file 3 — Additional file 3 Table S3. Positively selected SNPs identified in comparison of Middle East fat-tailed sheep vs European thin-tailed sheep. [file 12864_2020_7210_MOESM3_ESM.doc]

Table S3. Positively selected SNPs identified in comparison of Middle East fat-tailed sheep vs European thin-tailed sheep

| **CHR** | **SNP** | **Position** | **ancestralAllele** | **derivedAllele** | **ΔDAF** | **FST** |
| --- | --- | --- | --- | --- | --- | --- |
| 1 | s35058.1 | 6148701 | G | A | 0.341 | 0.324 |
| 1 | OAR1_9804499.1 | 10084179 | A | G | 0.341 | 0.312 |
| 1 | OAR1_66802799.1 | 63252034 | A | G | 0.463 | 0.347 |
| 1 | OAR1_66843581.1 | 63289944 | G | A | 0.427 | 0.311 |
| 1 | OAR1_71579102.1 | 66993139 | G | A | 0.528 | 0.462 |
| 1 | OAR1_71610348.1 | 67018785 | G | A | 0.362 | 0.302 |
| 1 | OAR1_89606616.1 | 84392176 | G | A | 0.417 | 0.334 |
| 1 | OAR1_100921673.1 | 94993712 | G | A | 0.560 | 0.476 |
| 1 | s20863.1 | 96216737 | A | G | 0.375 | 0.285 |
| 1 | OAR1_134233775.1 | 123854115 | A | G | 0.342 | 0.309 |
| 1 | s32216.1 | 128983286 | G | A | 0.366 | 0.318 |
| 1 | OAR1_165760275_X.1 | 154019699 | G | C | 0.376 | 0.303 |
| 1 | OAR1_183953098.1 | 170671157 | A | C | 0.361 | 0.299 |
| 1 | OAR1_183962708.1 | 170680226 | A | G | 0.371 | 0.308 |
| 1 | OAR1_208070059.1 | 192689940 | G | A | 0.336 | 0.317 |
| 1 | OAR1_208134575.1 | 192746782 | G | A | 0.345 | 0.285 |
| 1 | OAR1_253369226.1 | 234709908 | G | A | 0.364 | 0.286 |
| 1 | s02876.1 | 235155460 | G | A | 0.354 | 0.351 |
| 1 | OAR1_258547368.1 | 239162430 | G | A | 0.369 | 0.288 |
| 1 | s25871.1 | 244474339 | A | C | 0.361 | 0.307 |
| 1 | s73771.1 | 273653569 | A | G | 0.500 | 0.450 |
| 2 | OAR2_48310588_X.1 | 45500785 | A | G | 0.480 | 0.431 |
| 2 | OAR2_62874966.1 | 58497237 | C | G | 0.395 | 0.319 |
| 2 | OAR2_63072274.1 | 58698919 | G | A | 0.442 | 0.354 |
| 2 | OAR2_79499240.1 | 74561789 | C | A | 0.464 | 0.354 |
| 2 | OAR2_79550682.1 | 74619113 | G | A | 0.541 | 0.450 |
| 2 | OAR2_79594745.1 | 74665004 | A | G | 0.607 | 0.538 |
| 2 | s62643.1 | 74700075 | G | A | 0.567 | 0.491 |
| 2 | OAR2_83532585.1 | 78316809 | G | A | 0.374 | 0.300 |
| 2 | s66040.1 | 81746910 | G | A | 0.386 | 0.291 |
| 2 | OAR2_90617049.1 | 85339910 | A | G | 0.351 | 0.286 |
| 2 | s52970.1 | 86409485 | A | G | 0.508 | 0.412 |
| 2 | OAR2_95865952.1 | 89359755 | G | A | 0.364 | 0.282 |
| 2 | OAR2_113952627.1 | 105984105 | A | G | 0.412 | 0.324 |
| 2 | OAR2_129905238.1 | 121484489 | A | G | 0.397 | 0.322 |
| 2 | s75223.1 | 164494551 | G | A | 0.407 | 0.282 |
| 2 | s71934.1 | 175068089 | G | A | 0.377 | 0.305 |
| 2 | OAR2_192983948.1 | 182124730 | G | A | 0.364 | 0.289 |
| 2 | OAR2_193444466_X.1 | 182607165 | G | A | 0.478 | 0.403 |
| 2 | OAR2_194090177.1 | 183164212 | A | G | 0.637 | 0.582 |
| 2 | s12968.1 | 184725786 | G | A | 0.372 | 0.314 |
| 2 | s42892.1 | 184885519 | G | A | 0.344 | 0.283 |
| 2 | s14470.1 | 192231314 | A | G | 0.456 | 0.401 |
| 2 | OAR2_205216089.1 | 193669537 | G | A | 0.356 | 0.282 |
| 2 | OAR2_210261780.1 | 198596572 | A | G | 0.353 | 0.292 |
| 2 | OAR2_216769207.1 | 204761784 | G | A | 0.363 | 0.296 |
| 2 | OAR2_236955619.1 | 224283993 | G | A | 0.441 | 0.341 |
| 2 | OAR2_252554014.1 | 239141565 | G | A | 0.410 | 0.378 |
| 3 | s48427.1 | 26936637 | G | A | 0.391 | 0.313 |
| 3 | s16146.1 | 27024144 | G | A | 0.449 | 0.361 |
| 3 | OAR3_29169436.1 | 27076030 | A | G | 0.368 | 0.282 |
| 3 | OAR3_29696716.1 | 27430018 | G | A | 0.382 | 0.311 |
| 3 | OAR3_44619579.1 | 41701081 | G | A | 0.408 | 0.302 |
| 3 | OAR3_53590166.1 | 50888555 | A | G | 0.372 | 0.292 |
| 3 | OAR3_73718195.1 | 69775020 | G | A | 0.421 | 0.308 |
| 3 | OAR3_100019044.1 | 94148739 | G | A | 0.434 | 0.326 |
| 3 | OAR3_112264725.1 | 105585637 | G | A | 0.450 | 0.355 |
| 3 | OAR3_126893362.1 | 118982570 | A | G | 0.319 | 0.295 |
| 3 | OAR3_138290871.1 | 129648614 | G | A | 0.345 | 0.293 |
| 3 | OAR3_141586525.1 | 132478420 | G | A | 0.620 | 0.546 |
| 3 | OAR3_141703798.1 | 132549121 | G | A | 0.393 | 0.282 |
| 3 | s29248.1 | 132935814 | G | A | 0.403 | 0.379 |
| 3 | s68368.1 | 134234489 | G | A | 0.410 | 0.340 |
| 3 | s58504.1 | 134686259 | G | A | 0.371 | 0.293 |
| 3 | OAR3_183605277.1 | 171004273 | G | A | 0.329 | 0.310 |
| 3 | s07258.1 | 171544143 | G | A | 0.366 | 0.298 |
| 3 | OAR3_200805613.1 | 186593583 | A | G | 0.525 | 0.436 |
| 3 | OAR3_200820005_X.1 | 186613455 | G | A | 0.497 | 0.419 |
| 3 | OAR3_216333759.1 | 201094243 | G | A | 0.375 | 0.301 |
| 3 | s23020.1 | 213333467 | G | A | 0.355 | 0.307 |
| 3 | s34534.1 | 214673764 | C | A | 0.398 | 0.315 |
| 4 | OAR4_10580706.1 | 10435743 | A | G | 0.392 | 0.280 |
| 4 | OAR4_13728691.1 | 13533269 | G | A | 0.393 | 0.346 |
| 4 | s70608.1 | 14117077 | C | A | 0.383 | 0.388 |
| 4 | OAR4_27379611.1 | 26007577 | G | A | 0.376 | 0.311 |
| 4 | OAR4_27722499.1 | 26323779 | C | A | 0.451 | 0.345 |
| 4 | OAR4_28811142.1 | 27431427 | G | A | 0.462 | 0.369 |
| 4 | s04026.1 | 92688260 | A | G | 0.454 | 0.363 |
| 5 | s59159.1 | 12462150 | A | G | 0.342 | 0.302 |
| 5 | s33925.1 | 17890738 | C | A | 0.351 | 0.361 |
| 5 | s71344.1 | 41436761 | G | A | 0.482 | 0.399 |
| 5 | OAR5_47251900.1 | 43225641 | G | A | 0.512 | 0.411 |
| 5 | OAR5_47263230.1 | 43236671 | G | A | 0.579 | 0.537 |
| 6 | OAR6_13093410.1 | 10573000 | A | C | 0.402 | 0.301 |
| 6 | OAR6_40370293.1 | 36155169 | A | G | 0.433 | 0.332 |
| 6 | OAR6_41476497.1 | 37254883 | A | G | 0.420 | 0.381 |
| 6 | OAR6_41558126.1 | 37334387 | C | A | 0.412 | 0.366 |
| 6 | OAR6_41709987.1 | 37478366 | A | C | 0.396 | 0.405 |
| 6 | OAR6_41768532.1 | 37533664 | G | A | 0.514 | 0.455 |
| 6 | OAR6_44395905.1 | 39713559 | A | G | 0.373 | 0.299 |
| 6 | OAR6_58211072.1 | 52796762 | A | G | 0.358 | 0.294 |
| 6 | OAR6_73087918.1 | 66696744 | A | G | 0.392 | 0.290 |
| 7 | OAR7_1827930.1 | 2107907 | C | A | 0.389 | 0.311 |
| 7 | OAR7_30772408.1 | 27063794 | A | G | 0.320 | 0.280 |
| 7 | s13997.1 | 33988352 | G | A | 0.422 | 0.359 |
| 7 | OAR7_38284726.1 | 34080913 | C | G | 0.385 | 0.285 |
| 7 | OAR7_46765080.1 | 42133836 | G | A | 0.416 | 0.380 |
| 7 | OAR7_55546671.1 | 50172322 | G | A | 0.417 | 0.308 |
| 7 | OAR7_75664793_X.1 | 68996110 | A | G | 0.448 | 0.358 |
| 7 | OAR7_96995223.1 | 89156865 | G | A | 0.479 | 0.436 |
| 7 | OAR7_97009654.1 | 89171610 | A | C | 0.512 | 0.465 |
| 7 | OAR7_97127242.1 | 89263176 | A | G | 0.378 | 0.295 |
| 7 | OAR7_97159644.1 | 89286934 | G | A | 0.485 | 0.407 |
| 7 | OAR7_97629466.1 | 89758303 | G | A | 0.416 | 0.336 |
| 7 | s74641.1 | 98259681 | G | A | 0.366 | 0.288 |
| 7 | s20973.1 | 99181489 | G | A | 0.372 | 0.304 |
| 8 | OAR8_54864928.1 | 51164657 | G | A | 0.385 | 0.288 |
| 9 | s07939.1 | 30016843 | G | A | 0.351 | 0.284 |
| 9 | OAR9_33215449.1 | 31693511 | A | C | 0.371 | 0.283 |
| 9 | OAR9_55121048.1 | 52643203 | C | A | 0.390 | 0.320 |
| 9 | OAR9_55525366.1 | 53055485 | A | G | 0.365 | 0.307 |
| 9 | OAR9_70187264.1 | 66214217 | G | A | 0.422 | 0.361 |
| 9 | OAR9_99601991.1 | 93576726 | G | A | 0.580 | 0.515 |
| 10 | s45528.1 | 20551435 | G | A | 0.454 | 0.366 |
| 10 | OAR10_19917915.1 | 20584964 | G | A | 0.443 | 0.354 |
| 10 | OAR10_29312907_X.1 | 29276560 | G | A | 0.406 | 0.348 |
| 10 | s18834.1 | 29806294 | A | G | 0.406 | 0.365 |
| 10 | s68983.1 | 30618253 | G | A | 0.390 | 0.417 |
| 10 | s12004.1 | 30674002 | G | A | 0.358 | 0.419 |
| 10 | OAR10_30746533.1 | 30700853 | A | G | 0.385 | 0.354 |
| 11 | s63011.1 | 11443436 | A | C | 0.423 | 0.320 |
| 11 | s69909.1 | 18581219 | G | A | 0.482 | 0.435 |
| 11 | s45459.1 | 39292459 | G | A | 0.376 | 0.305 |
| 11 | OAR11_44391045.1 | 41763598 | A | G | 0.355 | 0.322 |
| 11 | OAR11_44415869.1 | 41788310 | A | G | 0.319 | 0.280 |
| 11 | s51300.1 | 51684772 | A | C | 0.395 | 0.297 |
| 11 | CZ923139_448.1 | 61111321 | G | A | 0.431 | 0.318 |
| 12 | s58825.1 | 3885444 | A | G | 0.392 | 0.326 |
| 12 | OAR12_11613774_X.1 | 9435445 | G | A | 0.380 | 0.292 |
| 12 | OAR12_24096729.1 | 20952545 | A | G | 0.485 | 0.370 |
| 12 | OAR12_24280419.1 | 21118437 | G | A | 0.420 | 0.332 |
| 12 | OAR12_40366545.1 | 36154610 | A | G | 0.503 | 0.431 |
| 12 | OAR12_70479451.1 | 63960016 | A | C | 0.360 | 0.309 |
| 12 | OAR12_72924306.1 | 66252130 | G | A | 0.472 | 0.442 |
| 13 | OAR13_38119417.1 | 34718056 | G | A | 0.380 | 0.325 |
| 13 | s08503.1 | 39890832 | G | A | 0.341 | 0.292 |
| 13 | s51138.1 | 47654121 | G | A | 0.494 | 0.476 |
| 13 | s60004.1 | 48303384 | A | G | 0.403 | 0.288 |
| 13 | OAR13_51852034.1 | 48623826 | G | A | 0.358 | 0.375 |
| 13 | OAR13_52180002.1 | 48768085 | A | G | 0.435 | 0.347 |
| 13 | s27419.1 | 48968332 | G | A | 0.448 | 0.464 |
| 13 | s67173.1 | 52779626 | A | G | 0.329 | 0.282 |
| 13 | s46007.1 | 53076283 | A | G | 0.373 | 0.292 |
| 13 | s46371.1 | 53111186 | A | G | 0.418 | 0.350 |
| 13 | s66957.1 | 53173647 | G | A | 0.426 | 0.357 |
| 13 | OAR13_82845711.1 | 76928813 | G | A | 0.392 | 0.327 |
| 14 | OAR14_36887906.1 | 35453538 | A | G | 0.495 | 0.400 |
| 14 | s36271.1 | 35607975 | A | G | 0.411 | 0.306 |
| 14 | OAR14_52322927.1 | 49643480 | G | A | 0.441 | 0.350 |
| 14 | s60143.1 | 51634626 | A | G | 0.490 | 0.432 |
| 15 | OAR15_1533836.1 | 1020813 | A | G | 0.335 | 0.282 |
| 15 | OAR15_2999185.1 | 3499482 | G | A | 0.460 | 0.380 |
| 15 | OAR15_3091174.1 | 3706790 | G | A | 0.589 | 0.539 |
| 15 | OAR15_5363781.1 | 5916755 | G | A | 0.394 | 0.302 |
| 15 | s13546.1 | 20337182 | A | G | 0.415 | 0.309 |
| 15 | OAR15_21252561.1 | 20362605 | A | G | 0.421 | 0.371 |
| 15 | OAR15_37561366.1 | 35721980 | G | A | 0.365 | 0.293 |
| 15 | OAR15_55184101.1 | 50486417 | G | A | 0.393 | 0.352 |
| 15 | s40044.1 | 53764196 | G | A | 0.332 | 0.331 |
| 15 | OAR15_80330932.1 | 74146128 | G | A | 0.427 | 0.316 |
| 16 | s31988.1 | 3359229 | A | G | 0.321 | 0.282 |
| 16 | OAR16_25179341.1 | 23091876 | A | G | 0.380 | 0.291 |
| 16 | OAR16_41804913.1 | 38538612 | G | A | 0.388 | 0.300 |
| 16 | OAR16_42321024.1 | 38952447 | A | G | 0.417 | 0.337 |
| 17 | OAR17_26494445.1 | 23944149 | A | G | 0.391 | 0.336 |
| 17 | OAR17_37138062.1 | 34121657 | C | G | 0.416 | 0.304 |
| 17 | s40690.1 | 44975901 | G | A | 0.402 | 0.328 |
| 17 | OAR17_58064576.1 | 53350131 | A | G | 0.498 | 0.409 |
| 17 | s09129.1 | 53470559 | A | G | 0.428 | 0.310 |
| 17 | s49475.1 | 53499969 | G | A | 0.452 | 0.341 |
| 17 | s18296.1 | 55710252 | C | A | 0.393 | 0.295 |
| 17 | s04192.1 | 62545448 | G | A | 0.399 | 0.290 |
| 17 | s75012.1 | 63851892 | A | C | 0.423 | 0.354 |
| 18 | s31152.1 | 19342316 | G | A | 0.391 | 0.422 |
| 18 | OAR18_22391503_X.1 | 21786173 | A | G | 0.426 | 0.331 |
| 18 | s42419.1 | 23552645 | G | A | 0.418 | 0.360 |
| 18 | s02246.1 | 32588588 | G | A | 0.341 | 0.307 |
| 18 | s32306.1 | 33162048 | G | A | 0.367 | 0.341 |
| 18 | s29190.1 | 33194278 | G | A | 0.348 | 0.287 |
| 18 | OAR18_38520499.1 | 36472813 | A | G | 0.364 | 0.296 |
| 18 | s10898.1 | 66358252 | A | G | 0.346 | 0.321 |
| 19 | OAR19_33278780.1 | 31599394 | A | G | 0.411 | 0.369 |
| 19 | s63765.1 | 45498763 | G | A | 0.471 | 0.361 |
| 19 | s18532.1 | 53198678 | A | G | 0.370 | 0.291 |
| 19 | s12882.1 | 54952611 | G | A | 0.385 | 0.320 |
| 21 | s57643.1 | 2505963 | G | A | 0.381 | 0.332 |
| 21 | OAR21_10702527.1 | 9308676 | A | G | 0.332 | 0.305 |
| 21 | OAR21_14592163.1 | 12911716 | G | A | 0.364 | 0.293 |
| 21 | OAR21_45227967.1 | 41106368 | C | A | 0.485 | 0.447 |
| 21 | s11631.1 | 41161582 | A | G | 0.452 | 0.428 |
| 21 | OAR21_50331592.1 | 45332006 | G | A | 0.410 | 0.334 |
| 23 | OAR23_32570581.1 | 30889404 | G | A | 0.385 | 0.315 |
| 23 | OAR23_58151044.1 | 54676534 | G | A | 0.422 | 0.382 |
| 23 | OAR23_66685354.1 | 62298841 | G | A | 0.386 | 0.330 |
| 24 | s48115.1 | 9865712 | A | G | 0.329 | 0.389 |
| 24 | s18520.1 | 11056039 | G | A | 0.335 | 0.280 |
| 25 | s14262.1 | 40285465 | G | A | 0.318 | 0.287 |
| 25 | s73888.1 | 43393194 | A | G | 0.382 | 0.296 |
| 25 | OAR25_47522993.1 | 44567090 | G | A | 0.361 | 0.291 |
| 26 | s13427.1 | 30781154 | A | C | 0.462 | 0.400 |
| 26 | s50766.1 | 34753094 | A | G | 0.455 | 0.381 |
| 26 | OAR26_46921474.1 | 41364432 | G | A | 0.401 | 0.297 |
