# Supplementary material for: Genomic analysis of worldwide sheep breeds reveals PDGFD as a major target of fat-tail selection in sheep
Source: BMC Genomics. 2020 Nov 17;21:800. doi: 10.1186/s12864-020-07210-9 (PMC7670677; doi:10.1186/s12864-020-07210-9)
Supplement: Supplementary file 4 — Additional file 4 Table S4. Positively selected SNPs identified in comparison of Chinese fat-tailed sheep vs South Asian thin-tailed sheep. [file 12864_2020_7210_MOESM4_ESM.doc]

Table S4. Positively selected SNPs identified in comparison of Chinese fat-tailed sheep vs South Asian thin-tailed sheep

| **CHR** | **SNP** | **Position** | **ancestralAllele** | **derivedAllele** | **ΔDAF** | **FST** |
| --- | --- | --- | --- | --- | --- | --- |
| 1 | OAR1_34027998.1 | 33295297 | A | G | 0.289 | 0.219 |
| 1 | OAR1_39449473.1 | 38247279 | A | C | 0.252 | 0.240 |
| 1 | s67807.1 | 54170411 | A | G | 0.252 | 0.232 |
| 1 | OAR1_77541835.1 | 72478142 | A | G | 0.305 | 0.248 |
| 1 | OAR1_100921673.1 | 94993712 | G | A | 0.485 | 0.382 |
| 1 | OAR1_102071635.1 | 95792924 | C | G | 0.337 | 0.233 |
| 1 | s09516.1 | 99470201 | A | G | 0.347 | 0.249 |
| 1 | s47856.1 | 105512098 | G | A | 0.368 | 0.258 |
| 1 | s23936.1 | 114785821 | G | A | 0.339 | 0.246 |
| 1 | OAR1_124203566.1 | 115232883 | G | A | 0.263 | 0.239 |
| 1 | s32216.1 | 128983286 | G | A | 0.415 | 0.313 |
| 1 | OAR1_175012572.1 | 162256804 | G | A | 0.321 | 0.226 |
| 1 | OAR1_217497923.1 | 201539484 | C | A | 0.331 | 0.237 |
| 1 | OAR1_268303279_X.1 | 248478013 | A | G | 0.271 | 0.214 |
| 1 | OAR1_270547664.1 | 250456371 | A | G | 0.270 | 0.226 |
| 1 | OAR1_276613221.1 | 256016538 | A | G | 0.284 | 0.227 |
| 2 | s36053.1 | 9157918 | A | G | 0.331 | 0.222 |
| 2 | OAR2_31412377.1 | 30492514 | G | A | 0.301 | 0.222 |
| 2 | s62463.1 | 72644072 | G | A | 0.303 | 0.263 |
| 2 | s00950.1 | 81689824 | G | A | 0.332 | 0.236 |
| 2 | OAR2_87543957.1 | 82346323 | G | A | 0.257 | 0.219 |
| 2 | DU314213_585.1 | 86465561 | G | A | 0.351 | 0.295 |
| 2 | s60772.1 | 148013217 | G | A | 0.344 | 0.216 |
| 2 | OAR2_193017597.1 | 182159410 | G | A | 0.399 | 0.323 |
| 2 | OAR2_193444466_X.1 | 182607165 | G | A | 0.363 | 0.358 |
| 2 | OAR2_195165011.1 | 184070090 | A | G | 0.281 | 0.255 |
| 2 | s48702.1 | 219847222 | G | A | 0.271 | 0.253 |
| 2 | OAR2_236955619.1 | 224283993 | G | A | 0.445 | 0.379 |
| 2 | OAR2_239847244.1 | 227091775 | G | A | 0.274 | 0.226 |
| 2 | s37310.1 | 232414241 | A | T | 0.280 | 0.234 |
| 3 | OAR3_43027143.1 | 40285089 | A | C | 0.385 | 0.253 |
| 3 | OAR3_44467884.1 | 41551185 | A | G | 0.437 | 0.357 |
| 3 | s25321.1 | 62015360 | G | A | 0.419 | 0.371 |
| 3 | s68356.1 | 62044832 | A | G | 0.460 | 0.400 |
| 3 | OAR3_71832206.1 | 67973248 | G | A | 0.336 | 0.226 |
| 3 | s12614.1 | 74650966 | A | G | 0.316 | 0.294 |
| 3 | OAR3_83483148.1 | 78930206 | G | A | 0.351 | 0.225 |
| 3 | s04306.1 | 79315264 | G | A | 0.284 | 0.217 |
| 3 | s22567.1 | 81989671 | C | A | 0.425 | 0.355 |
| 3 | s22632.1 | 82049915 | A | G | 0.474 | 0.412 |
| 3 | s49870.1 | 99660554 | G | A | 0.369 | 0.232 |
| 3 | OAR3_114225433.1 | 107334356 | A | G | 0.298 | 0.267 |
| 3 | OAR3_126195855.1 | 118325231 | G | A | 0.417 | 0.342 |
| 3 | OAR3_126893362.1 | 118982570 | A | G | 0.307 | 0.290 |
| 3 | OAR3_138052548_X.1 | 129423378 | G | A | 0.326 | 0.230 |
| 3 | s71169.1 | 129759745 | C | A | 0.466 | 0.388 |
| 3 | OAR3_141586525.1 | 132478420 | G | A | 0.310 | 0.236 |
| 3 | s50426.1 | 133886147 | G | A | 0.310 | 0.223 |
| 3 | s53492.1 | 134161083 | G | A | 0.310 | 0.320 |
| 3 | s31828.1 | 134177731 | G | A | 0.324 | 0.308 |
| 3 | OAR3_164047094.1 | 153388484 | A | G | 0.337 | 0.242 |
| 3 | OAR3_165050963.1 | 154252449 | G | A | 0.282 | 0.252 |
| 3 | OAR3_183846062.1 | 171249010 | A | C | 0.258 | 0.226 |
| 3 | OAR3_187883913.1 | 175057434 | A | G | 0.267 | 0.274 |
| 3 | s42143.1 | 180634526 | A | G | 0.388 | 0.251 |
| 3 | OAR3_200125923.1 | 185881136 | A | G | 0.332 | 0.246 |
| 3 | OAR3_204157824.1 | 189580068 | G | A | 0.403 | 0.291 |
| 3 | s24239.1 | 218572118 | G | A | 0.304 | 0.226 |
| 4 | s25289.1 | 68802676 | A | G | 0.475 | 0.372 |
| 4 | s21178.1 | 98791243 | A | G | 0.376 | 0.265 |
| 5 | s23842.1 | 37078919 | A | G | 0.300 | 0.225 |
| 5 | s33002.1 | 48043887 | G | A | 0.280 | 0.267 |
| 5 | s69230.1 | 53764208 | A | C | 0.318 | 0.228 |
| 6 | OAR6_21601091.1 | 18643578 | G | A | 0.351 | 0.255 |
| 6 | OAR6_33994293.1 | 30173410 | A | G | 0.292 | 0.233 |
| 6 | OAR6_34010746.1 | 30188257 | A | G | 0.281 | 0.214 |
| 6 | OAR6_36667497.1 | 32594843 | G | A | 0.321 | 0.240 |
| 6 | OAR6_40370293.1 | 36155169 | A | G | 0.358 | 0.308 |
| 6 | OAR6_92321965.1 | 84350628 | G | A | 0.415 | 0.356 |
| 7 | OAR7_24921584.1 | 23778602 | A | G | 0.346 | 0.240 |
| 7 | OAR7_43027191.1 | 38944126 | A | G | 0.293 | 0.227 |
| 7 | s06699.1 | 64223678 | A | C | 0.321 | 0.221 |
| 7 | s08218.1 | 82488409 | A | G | 0.452 | 0.338 |
| 7 | s55494.1 | 82545552 | G | A | 0.611 | 0.594 |
| 7 | OAR7_97159644.1 | 89286934 | G | A | 0.318 | 0.221 |
| 8 | OAR8_18319939.1 | 16445482 | A | G | 0.339 | 0.233 |
| 8 | OAR8_55890096.1 | 52047484 | A | G | 0.334 | 0.226 |
| 8 | OAR8_58028748.1 | 54142002 | A | G | 0.308 | 0.243 |
| 8 | OAR8_60234503.1 | 56216655 | G | A | 0.338 | 0.264 |
| 8 | OAR8_68953183.1 | 64089140 | G | A | 0.348 | 0.269 |
| 8 | OAR8_74597457.1 | 69566431 | A | C | 0.358 | 0.236 |
| 8 | OARUn.2258_7828.1 | 82331653 | A | T | 0.316 | 0.304 |
| 8 | s63980.1 | 87826427 | A | G | 0.431 | 0.357 |
| 9 | OAR9_38099807.1 | 36163638 | A | G | 0.351 | 0.236 |
| 9 | OAR9_99601991.1 | 93576726 | G | A | 0.379 | 0.285 |
| 10 | OAR10_29538398.1 | 29502667 | G | A | 0.357 | 0.331 |
| 10 | s66464.1 | 35981007 | G | A | 0.362 | 0.228 |
| 10 | OAR10_36746264.1 | 35992688 | C | A | 0.355 | 0.224 |
| 10 | OAR10_36819129.1 | 36064927 | G | A | 0.357 | 0.229 |
| 10 | OAR10_89184637.1 | 81648118 | A | G | 0.336 | 0.222 |
| 11 | s50820.1 | 8911693 | G | A | 0.308 | 0.242 |
| 11 | OAR11_18701428.1 | 18325488 | G | A | 0.620 | 0.618 |
| 11 | OAR11_18815864.1 | 18433474 | G | A | 0.575 | 0.570 |
| 11 | OAR11_18823250.1 | 18440783 | A | G | 0.534 | 0.498 |
| 11 | OAR11_19810690.1 | 19407955 | G | A | 0.369 | 0.299 |
| 12 | OAR12_27046622.1 | 23608111 | C | G | 0.408 | 0.375 |
| 12 | OAR12_28880865.1 | 25398197 | G | A | 0.347 | 0.227 |
| 12 | OAR12_35168968.1 | 31357766 | G | A | 0.293 | 0.229 |
| 12 | OAR12_64690849.1 | 58309490 | G | A | 0.328 | 0.237 |
| 12 | OAR12_72924306.1 | 66252130 | G | A | 0.380 | 0.354 |
| 13 | DU435573_466.1 | 30047921 | G | A | 0.383 | 0.294 |
| 13 | s45399.1 | 34613483 | A | G | 0.325 | 0.217 |
| 13 | OAR13_47815459.1 | 44477118 | G | A | 0.364 | 0.267 |
| 13 | s51138.1 | 47654121 | G | A | 0.346 | 0.240 |
| 13 | OAR13_51492534.1 | 48264810 | A | G | 0.307 | 0.219 |
| 13 | OAR13_51727898.1 | 48493120 | A | C | 0.589 | 0.530 |
| 13 | s40901.1 | 48553033 | A | G | 0.505 | 0.409 |
| 13 | OAR13_51817610.1 | 48585628 | C | A | 0.484 | 0.382 |
| 13 | OAR13_51852034.1 | 48623826 | G | A | 0.581 | 0.521 |
| 13 | OAR13_51886803.1 | 48657027 | C | A | 0.448 | 0.346 |
| 13 | s27419.1 | 48968332 | G | A | 0.773 | 0.759 |
| 13 | OAR13_53493947.1 | 50020891 | A | G | 0.288 | 0.222 |
| 14 | s43948.1 | 10834307 | G | A | 0.379 | 0.259 |
| 14 | OAR14_32509198.1 | 31290127 | G | A | 0.347 | 0.227 |
| 14 | OAR14_36887906.1 | 35453538 | A | G | 0.342 | 0.234 |
| 14 | s36271.1 | 35607975 | A | G | 0.253 | 0.216 |
| 15 | OAR15_2799595.1 | 3382802 | G | A | 0.368 | 0.241 |
| 15 | OAR15_2886961.1 | 3462742 | A | G | 0.484 | 0.382 |
| 15 | OAR15_2999185.1 | 3499482 | G | A | 0.490 | 0.426 |
| 15 | OARUn.1355_45411.1 | 3575948 | A | G | 0.550 | 0.474 |
| 15 | OAR15_3091174.1 | 3706790 | G | A | 0.820 | 0.788 |
| 15 | OAR15_3164045.1 | 3805470 | G | A | 0.467 | 0.398 |
| 15 | OAR15_3236575.1 | 3875564 | C | A | 0.388 | 0.316 |
| 15 | s28843.1 | 21615126 | G | A | 0.337 | 0.226 |
| 15 | s18566.1 | 54478418 | G | A | 0.270 | 0.244 |
| 15 | s21547.1 | 73321768 | A | C | 0.321 | 0.241 |
| 16 | s58695.1 | 11807075 | G | A | 0.383 | 0.306 |
| 16 | OAR16_41804913.1 | 38538612 | G | A | 0.352 | 0.289 |
| 16 | s50768.1 | 57094224 | G | A | 0.305 | 0.254 |
| 17 | OAR17_31561091.1 | 28790576 | A | G | 0.268 | 0.263 |
| 17 | OAR17_33260430.1 | 30423316 | A | G | 0.424 | 0.401 |
| 17 | OAR17_36966397.1 | 33954083 | A | G | 0.486 | 0.382 |
| 17 | s60720.1 | 34661256 | A | C | 0.307 | 0.251 |
| 17 | s55140.1 | 34801425 | C | G | 0.312 | 0.279 |
| 17 | OAR17_38506788.1 | 35482296 | A | G | 0.334 | 0.261 |
| 17 | OAR17_58008141.1 | 53294271 | A | C | 0.341 | 0.220 |
| 17 | OAR17_58064576.1 | 53350131 | A | G | 0.353 | 0.281 |
| 18 | s31152.1 | 19342316 | G | A | 0.255 | 0.257 |
| 19 | s38567.1 | 7169893 | G | A | 0.315 | 0.261 |
| 19 | OAR19_33605872.1 | 31927501 | A | G | 0.329 | 0.253 |
| 19 | OAR19_35971670.1 | 34228061 | A | G | 0.283 | 0.220 |
| 20 | OAR20_4842571.1 | 4835005 | A | G | 0.433 | 0.314 |
| 20 | s11944.1 | 9594109 | G | A | 0.322 | 0.280 |
| 20 | s39515.1 | 34256995 | G | A | 0.256 | 0.227 |
| 21 | s13077.1 | 33404625 | A | G | 0.288 | 0.234 |
| 22 | OAR22_10852834.1 | 9079941 | A | G | 0.379 | 0.324 |
| 22 | s00078.1 | 30355910 | G | A | 0.298 | 0.218 |
| 22 | s19503.1 | 36806429 | G | A | 0.413 | 0.319 |
| 22 | s19461.1 | 39739539 | G | A | 0.279 | 0.260 |
| 22 | s02448.1 | 40450104 | C | A | 0.294 | 0.226 |
| 22 | OAR22_45509727.1 | 40482146 | G | A | 0.408 | 0.347 |
| 22 | s28086.1 | 50505055 | G | A | 0.324 | 0.228 |
| 24 | s08464.1 | 35991517 | G | A | 0.328 | 0.332 |
| 24 | s34514.1 | 36036418 | G | A | 0.330 | 0.237 |
| 25 | OAR25_2326490.1 | 3273992 | A | G | 0.363 | 0.296 |
| 25 | s21601.1 | 38710382 | G | A | 0.502 | 0.395 |
| 26 | OAR26_44586910.1 | 39385787 | A | G | 0.343 | 0.277 |
