# Supplementary figures and images for: Genomic analysis of worldwide sheep breeds reveals PDGFD as a major target of fat-tail selection in sheep
Source: BMC Genomics. 2020 Nov 17;21:800. doi: 10.1186/s12864-020-07210-9 (PMC7670677; doi:10.1186/s12864-020-07210-9)

Figure S1

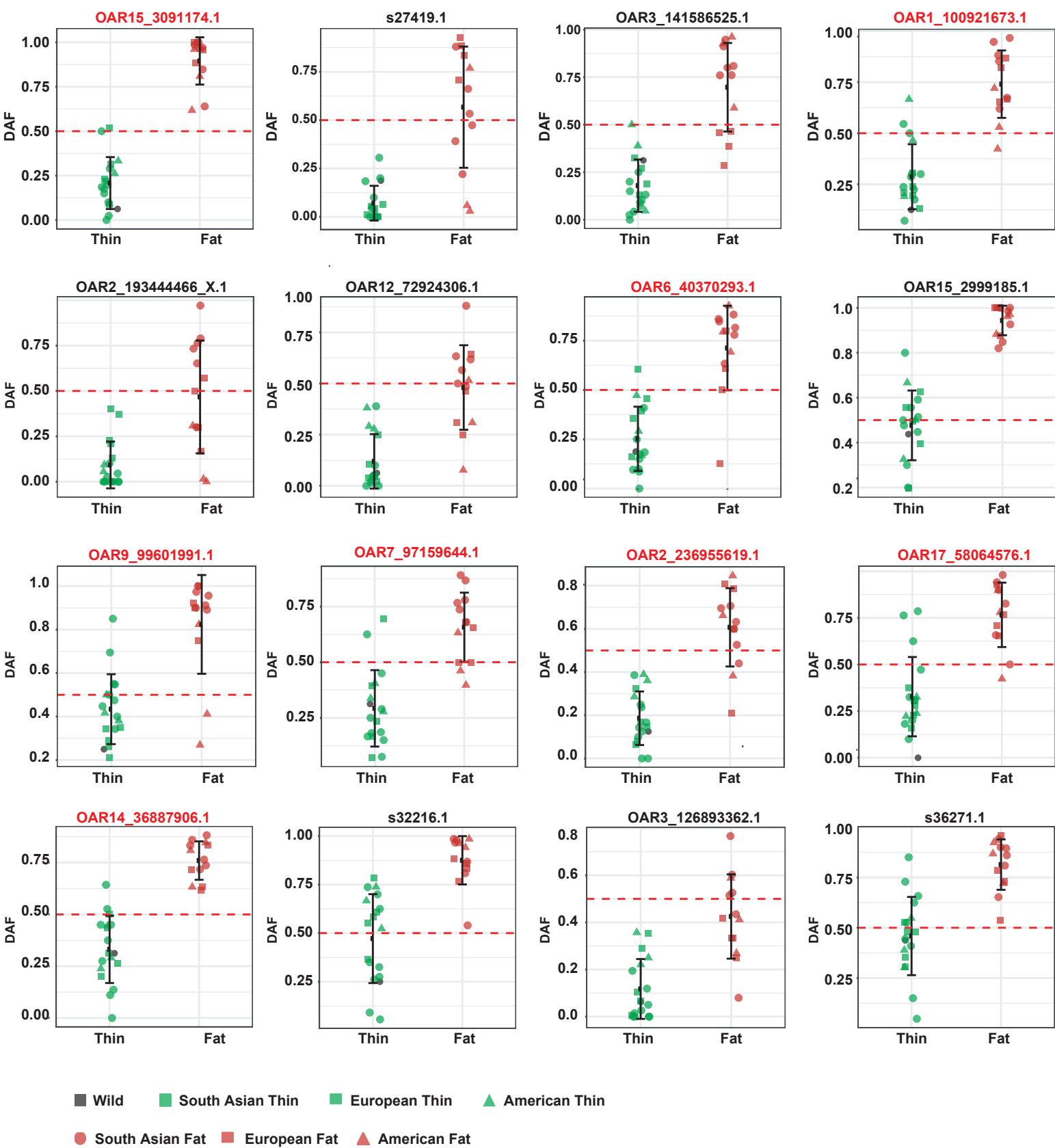

Supplement: Supplementary file 5 — Additional file 5 Figure S1. The distribution of derived allele frequency (DAF) of the 16 candidate SNPs in each breed. SNPs were sorted according to the average value of FST and ΔDAF among the three comparisons indicated in Fig. 1b from high to low. SNPs labeled in red represented promising candidate SNPs for fat tail in sheep and the remaining eight SNPs were excluded from the promising candidate list because they failed to pass the filter criterial described in Methods. [file 12864_2020_7210_MOESM5_ESM.pdf]

Figure S2

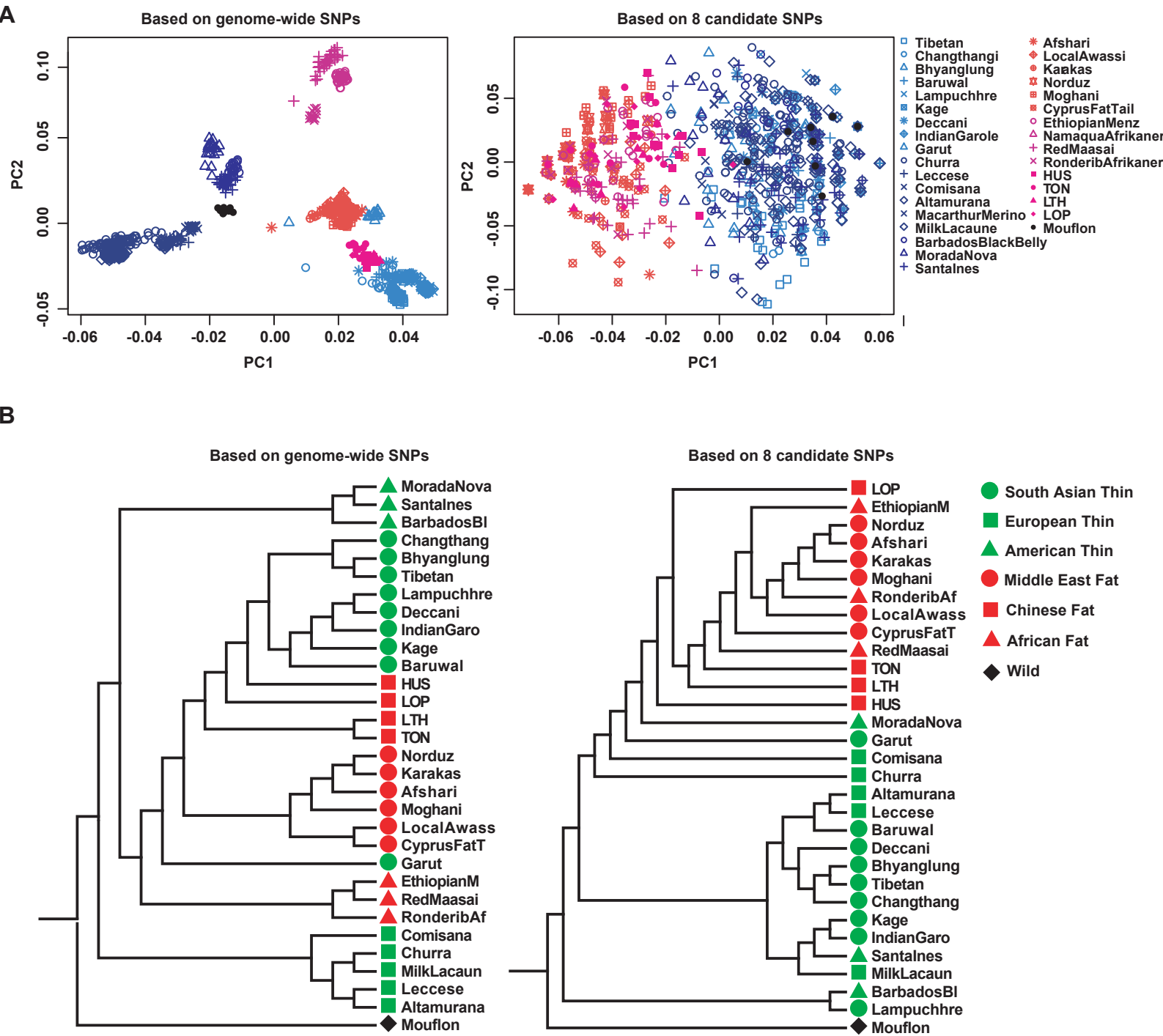

Supplement: Supplementary file 6 — Additional file 6 Figure S2. Phylogenetic analysis of the studied sheep breeds. (A) Principle Component Analysis (PCA) results. The left panel shows the PCA plot generated based on genome wide SNPs. The right panel shows the PCA plot generated based on the 8 candidate SNPs. (B) Phylogenetic tree results. The left panel shows the phylogenetic tree constructed according to genome wide SNPs. The right panel shows the phylogenetic tree constructed according to the 8 candidate SNPs. [file 12864_2020_7210_MOESM6_ESM.pdf]

Figure S3

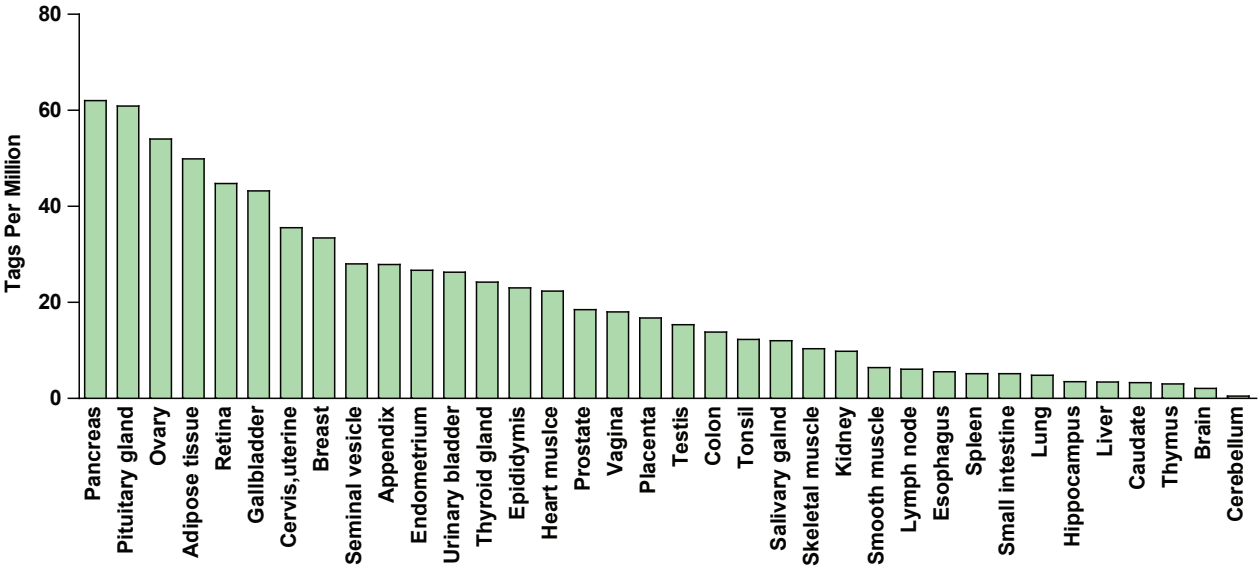

Supplement: Supplementary file 8 — Additional file 8 Figure S3. The expression level across different tissues of PDGFD gene obtained from FANTOM5 dataset from The Human Protein Atlas database. [file 12864_2020_7210_MOESM8_ESM.pdf]

Figure S5

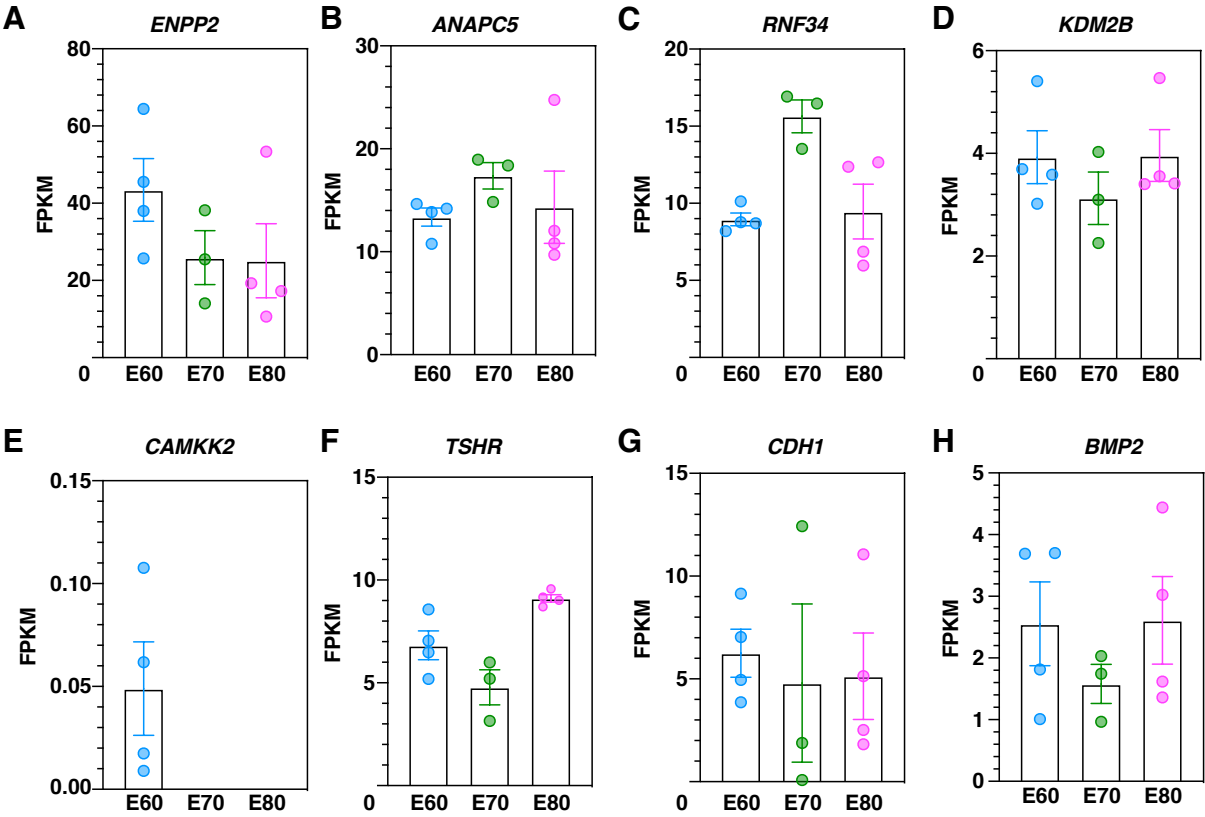

Supplement: Supplementary file 11 — Additional file 11 Figure S5. Expression of the top candidate genes in tail tissues of fat-tailed sheep during embryonic development revealed by RNA-seq. E60: embryonic day 60. E70: embryonic day 70. E80: embryonic day 80. [file 12864_2020_7210_MOESM11_ESM.pdf]
