# Supplementary material for: Genomic analysis of worldwide sheep breeds reveals PDGFD as a major target of fat-tail selection in sheep
Source: BMC Genomics. 2020 Nov 17;21:800. doi: 10.1186/s12864-020-07210-9 (PMC7670677; doi:10.1186/s12864-020-07210-9)
Supplement: Supplementary file 7 — Additional file 7 Table S5. Information of samples used for genome sequence variation analysis. [file 12864_2020_7210_MOESM7_ESM.doc]

Table S5. Information of samples used for genome sequence variation analysis

| **Smaple ID** | **Population** | **Origin** | **Tail type** |
| --- | --- | --- | --- |
| OARI_ZB08 | Tibetan | Asian | Thin |
| OARI_ZD11 | Tibetan | Asian | Thin |
| OARI_CHA02 | Changthangi | Asian | Thin |
| OARI_CHA05 | Changthangi | Asian | Thin |
| OARI_GAR4 | IndiaGarot | Asian | Thin |
| OARI_GAR14 | Garole | Asian | Thin |
| OARI_GUR4 | Garut | Asian | Thin |
| OARI_GUR5 | Garut | Asian | Thin |
| OARI_CHU1 | Churra | Europe | Thin |
| OARI_CHU2 | Churra | Europe | Thin |
| OARI_LAC1 | Meat Lacaune | Europe | Thin |
| OARI_LAC84 | Mmilk Lacaune | Europe | Thin |
| OARI_BMN3 | Morada Nova | Americas | Thin |
| OARI_BMN4 | Morada Nova | Americas | Thin |
| OARI_BSI3 | Santa Inês | Americas | Thin |
| OARI_BSI4 | Santa Inês | Americas | Thin |
| OARI_AFS32 | Afshari | MiddleEast | Fat |
| OARI_AFS33 | Afshari | MiddleEast | Fat |
| OARI_AW454 | LocalAwassi | MiddleEast | Fat |
| OARI_AWT1 | LocalAwassi | MiddleEast | Fat |
| OARI_AWT2 | LocalAwassi | MiddleEast | Fat |
| OARI_KRS3 | Karakas | MiddleEast | Fat |
| OARI_KRS5 | Karakas | MiddleEast | Fat |
| OARI_NDZ1 | Norduz | MiddleEast | Fat |
| OARI_NDZ4 | Norduz | MiddleEast | Fat |
| OARI_EMZ1 | EthiopianMenz | Africa | Fat |
| OARI_NQA11 | Namaqua Afrikaner | Africa | Fat |
| OARI_RDA2 | Ronderib Afrikaner | Africa | Fat |
| OARI_RDA4 | Ronderib Afrikaner | Africa | Fat |
