# Supplementary material for: Genomic analysis of worldwide sheep breeds reveals PDGFD as a major target of fat-tail selection in sheep
Source: BMC Genomics. 2020 Nov 17;21:800. doi: 10.1186/s12864-020-07210-9 (PMC7670677; doi:10.1186/s12864-020-07210-9)

**Table S6. Highly differentiated SNPs (ΔAF >0.60) within the putative targeted region of positive selection in *PDGFD* gene (Chr15:3,854,063-3,860,894 bp)**

| **SNP** | **Position** | **ΔAF** |
| --- | --- | --- |
| ss1139270974,ss1198779082 | 3854063 | 0.65 |
| ss1139270977 | 3854426 | 0.62789 |
| ss1139270978,ss1215832225,ss1198779102 | 3854617 | 0.75245 |
| ss1139270979,ss1215832228,ss1198779106 | 3854930 | 0.84375 |
| ss1139270980,ss1215832229,ss1198779108 | 3854933 | 0.84375 |
| ss1139270981,ss1215832230,ss1198779109 | 3854955 | 0.73558 |
| ss1139270982,ss1215832231,ss1198779113 | 3855064 | 0.81495 |
| ss1139270984,ss1215832233,ss1198779118 | 3855194 | 0.81495 |
| ss1139270986,ss1215832236,ss1198779122 | 3855324 | 0.81495 |
| ss1139270987,ss1215832237,ss1198779126 | 3855347 | 0.61058 |
| ss1139270990,ss1215832240,ss1198779129 | 3855390 | 0.81495 |
| ss1139270991,ss1215832242,ss1198779131 | 3855407 | 0.61058 |
| ss1139270992,ss1215832243,ss1198779137 | 3855530 | 0.6676 |
| ss1139270994,ss1215832245,ss1198779144 | 3855601 | 0.73558 |
| ss1215832247,ss1198779147 | 3855636 | 0.73558 |
| ss1215832252,ss1198779153 | 3855829 | 0.73558 |
| ss1139270997,ss1215832253,ss1198779156 | 3855847 | 0.69 |
| ss1139270998,ss1215832255,ss1198779163 | 3855934 | 0.69 |
| ss1139271000,ss1215832258 | 3856143 | 0.61058 |
| ss1139271002,ss1215832259,ss1198779172 | 3856224 | 0.72308 |
| ss1215832261,ss1198779175 | 3856258 | 0.73558 |
| ss1215832262,ss1198779177 | 3856263 | 0.69 |
| ss1139271004,ss1215832263,ss1198779181 | 3856492 | 0.81495 |
| ss1139271005,ss1215832268,ss1198779184 | 3856682 | 0.67308 |
| ss1215832274,ss1198779195 | 3857032 | 0.82935 |
| ss1215832275,ss1198779196 | 3857037 | 0.82935 |
| ss1215832276,ss1198779198 | 3857065 | 0.82935 |
| ss1139271006,ss1198779203 | 3857203 | 0.81495 |
| ss1215832277,ss1198779205 | 3857217 | 0.82935 |
| ss1215832282,ss1198779213 | 3857345 | 0.82935 |
| ss1139271007,ss1215832283 | 3857377 | 0.67308 |
| ss1139271008,ss1215832284 | 3857378 | 0.67308 |
| ss1215832286,ss1198779218 | 3857464 | 0.82935 |
| ss1215832288,ss1198779220 | 3857494 | 0.82935 |
| ss1139271011,ss1215832296,ss1198779236 | 3857817 | 0.7981 |
| ss1139271012,ss1215832297,ss1198779237 | 3857841 | 0.7981 |
| ss1139271013,ss1215832298,ss1198779239 | 3857900 | 0.7981 |
| ss1139271014,ss1215832299,ss1198779241 | 3857938 | 0.7981 |
| ss1139271016,ss1198779258 | 3858241 | 0.7981 |
| ss1139271017,ss1215832305,ss1198779259 | 3858247 | 0.7981 |
| ss1198779264 | 3858309 | 0.7981 |
| ss1139271019,ss1215832309,ss1198779270 | 3858370 | 0.7981 |
| ss1139271024,ss1215832322,ss1198779290 | 3858966 | 0.81495 |
| ss1139271031,ss1215832331,ss1198779312 | 3859314 | 0.81495 |
| ss1139271040,ss1215832345,ss1198779342 | 3860194 | 0.6587 |
| ss1139271042,ss1215832347,ss1198779345 | 3860228 | 0.81495 |
| ss1215832355,ss1198 ss1139270980,ss1215832229,ss1198779108779360 | 3860577 | 0.73558 |
| ss1139271048,ss1198779365 | 3860662 | 0.70428 |
| ss1215832357 | 3860685 | 0.8606 |
| ss1215832359,ss1198779374 | 3860771 | 0.85167 |
| ss1215832363 | 3860894 | 0.81495 |

Note: SNPs with red color indicate SNPs that were validated in expanded samples with Sequenom MassArray.

**Supplementary figures**

**Figure S1. The distribution of derived allele frequency (DAF) of the 16 candidate SNPs in each breed.** SNPs were sorted according to the average value of FST and ΔDAF among the three comparisons indicated in Figure 1b from high to low. SNPs labeled in red represented promising candidate SNPs for fat tail in sheep and the remaining eight SNPs were excluded from the promising candidate list because they failed to pass the filter criterial described in Methods.

**Figure S2. Phylogenetic analysis of the studied sheep breeds. (A)** Principle Component Analysis (PCA) results. The left panel shows the PCA plot generated based on genome wide SNPs. The right panel shows the PCA plot generated based on the 8 candidate SNPs. **(B)** Phylogenetic tree results. The left panel shows the phylogenetic tree constructed according to genome wide SNPs. The right panel shows the phylogenetic tree constructed according to the 8 candidate SNPs.

**Figure S3. The expression level across different tissues of *PDGFD* gene obtained from FANTOM5 dataset from The Human Protein Atlas database.**

**Figure S4. Replication of the top candidate SNPs proposed by three previous studies in all sheep breeds used in this study.** The top panel indicates the the derived allele frequency (DAF) of 10 top SNPs on chromosome 5 and 7 proposed by Moradi et al. (Table 3 in their study). The s553221.1 locus on chromosome 5 in their study was not included because it did not pass the quality control in this study. The middle panel shows the DAF of four SNPs corresponding to BMP2 and two SNPs corresponding to VRTN gene proposed by Moioli et al. (Table 2 in their study). The s73063.1 locus annotated to VRTN was not was not included because it did not pass the quality control in this study. The bottom panel represents the DAF of SNPs annotated to the key genes proposed by Yuan et al. The OAR4_73050615.1 locus is from Table 1 and other loci are from table 2 in their study.

**Figure S5. Expression of the top candidate genes in tail tissues of fat-tailed sheep during embryonic development revealed by RNA-seq.** E60: embryonic day 60. E70: embryonic day 80. E80: embryonic day 80.

**Figure S1**

**
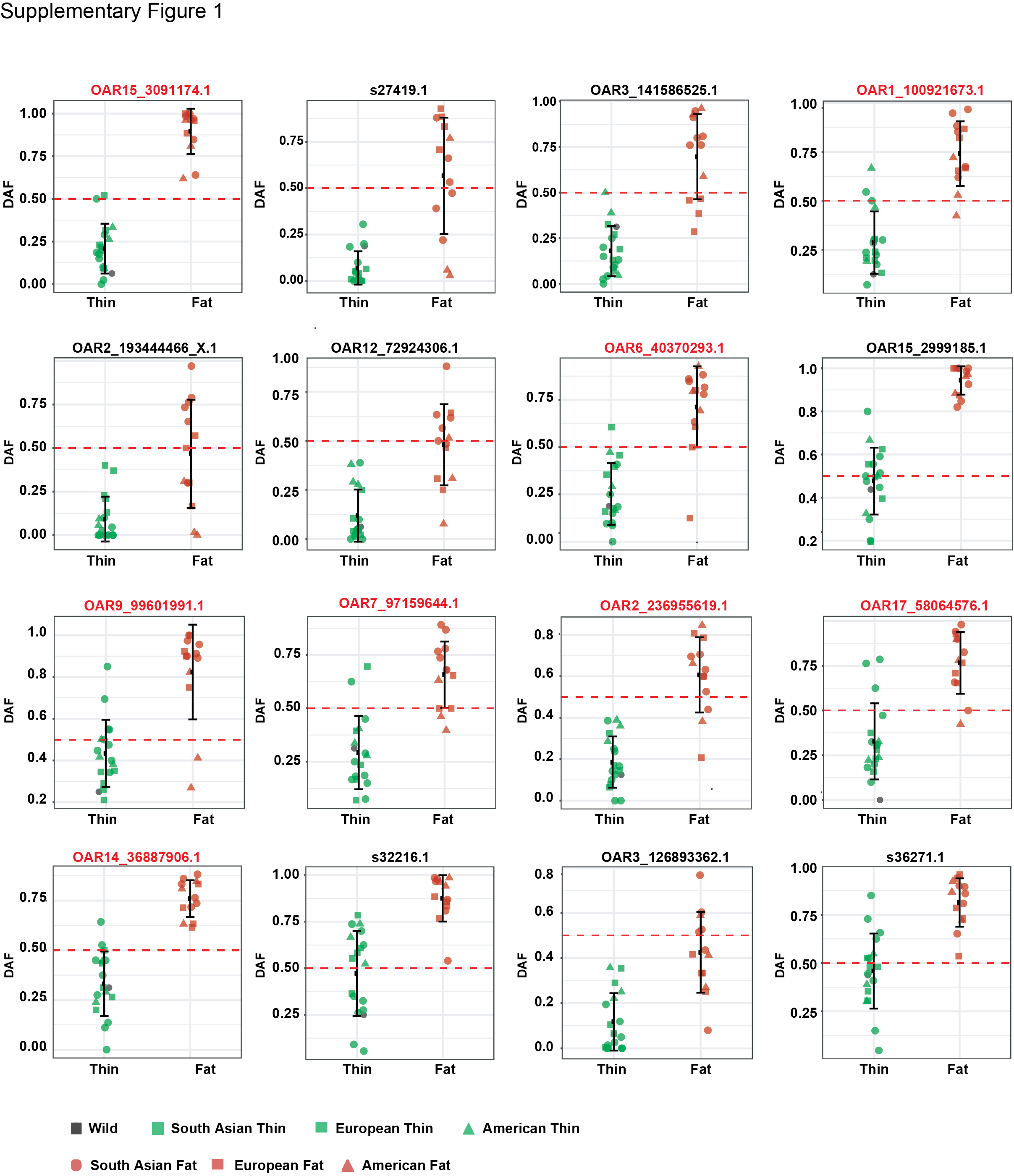
**

**Figure S2**


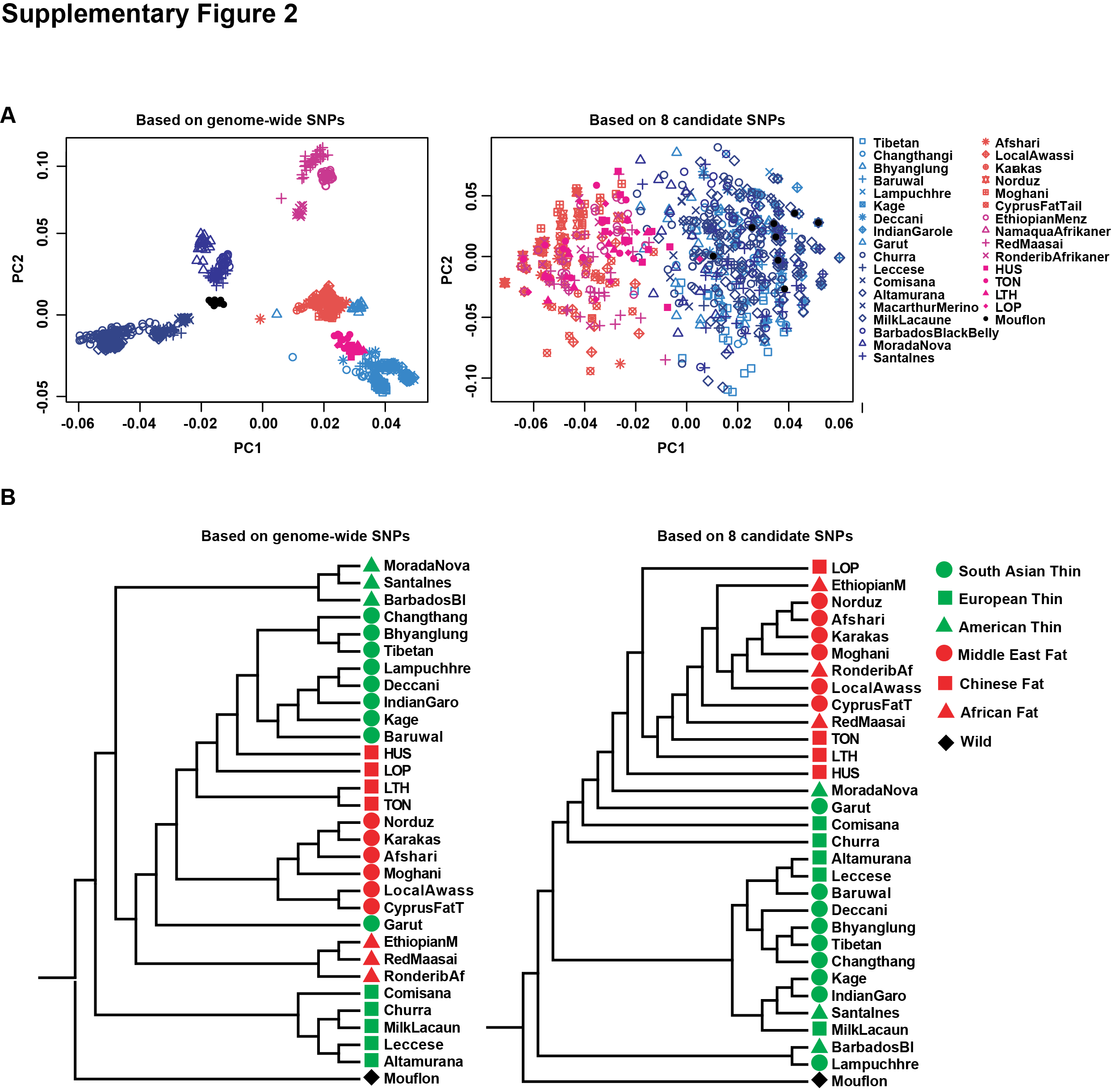


**Figure S3**


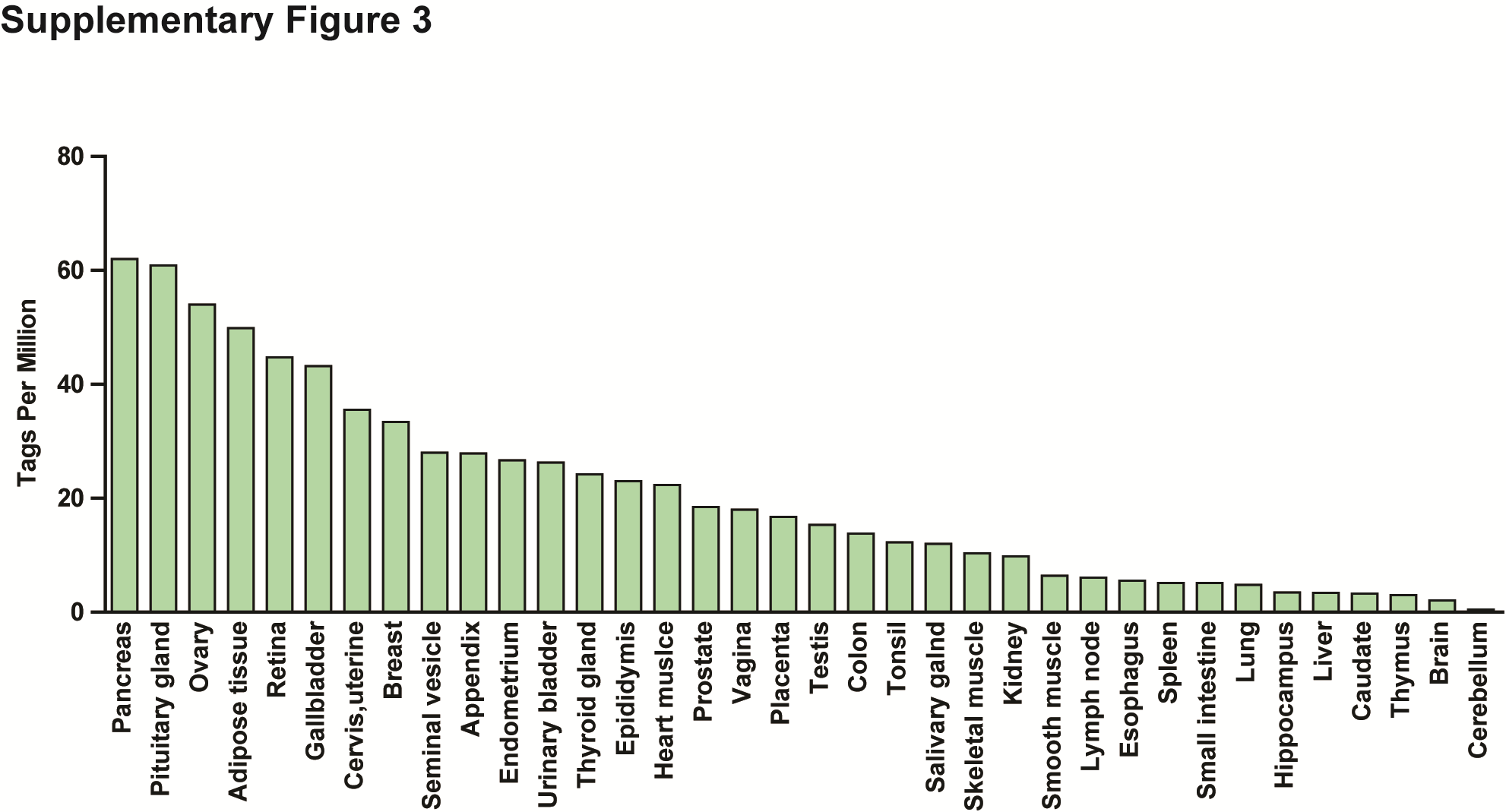


**Figure S4**


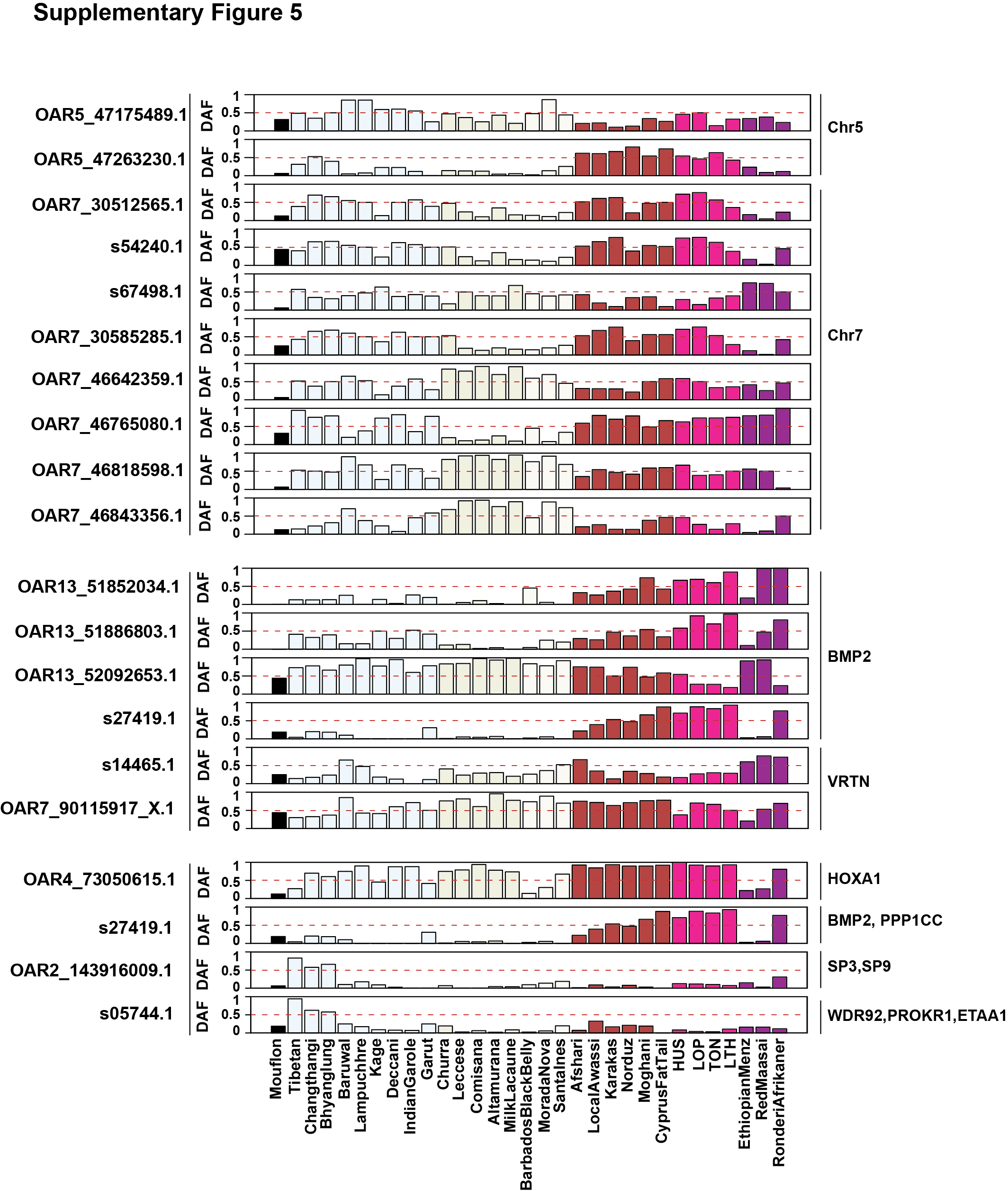


Figure S5


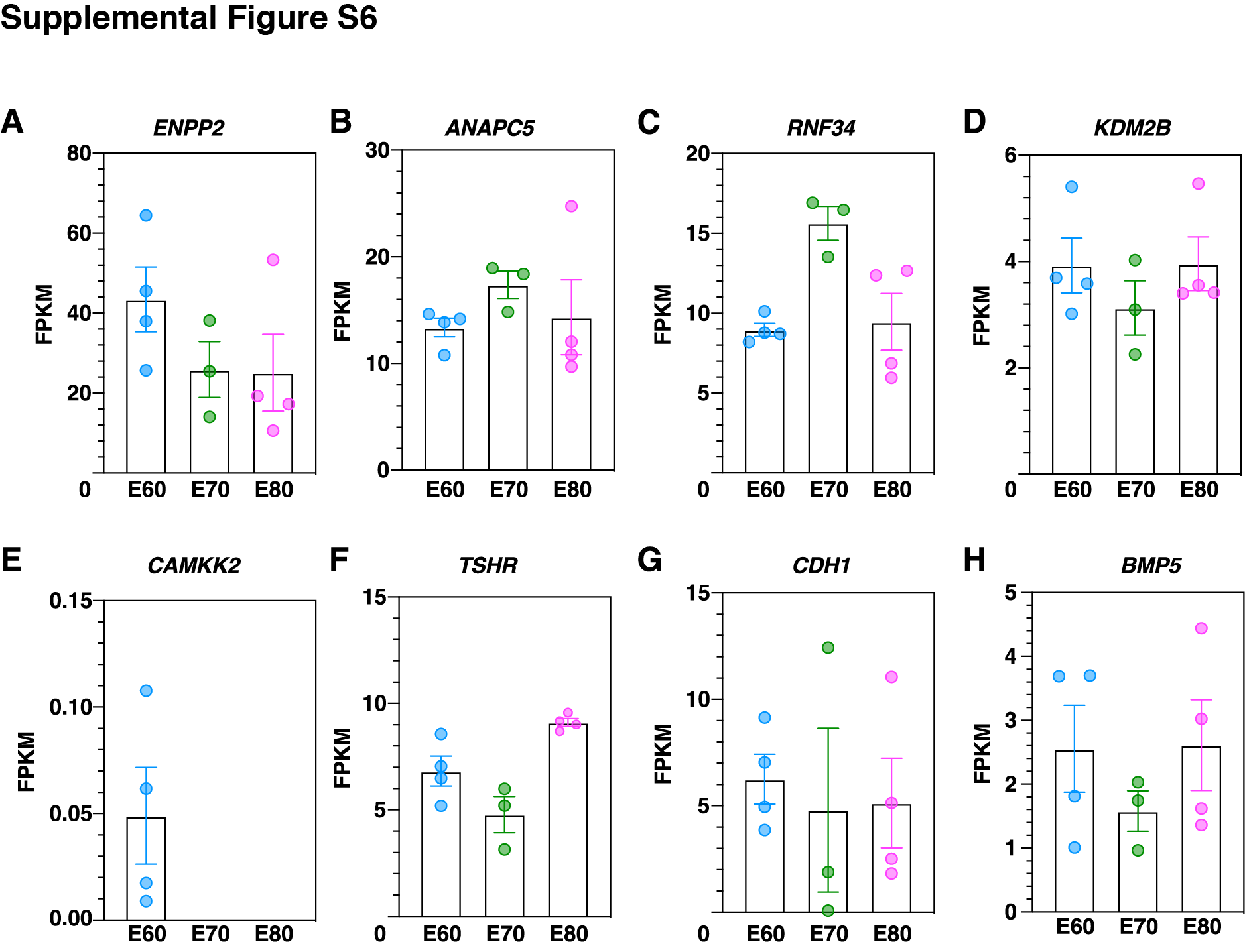

Supplement: Supplementary file 9 — Additional file 9 Table S6. Highly differentiated SNPs (ΔAF > 0.60) within the putative targeted region of positive selection in PDGFD gene (Chr15:3,854,063-3,860,894 bp). [file 12864_2020_7210_MOESM9_ESM.doc]
