# Supplementary material for: Genomic analysis of worldwide sheep breeds reveals PDGFD as a major target of fat-tail selection in sheep
Source: BMC Genomics. 2020 Nov 17;21:800. doi: 10.1186/s12864-020-07210-9 (PMC7670677; doi:10.1186/s12864-020-07210-9)
Supplement: Supplementary file 10 — Additional file 10 Figure S4. Replication of the top candidate SNPs proposed by three previous studies in all sheep breeds used in this study. (A) Derived allele frequency (DAF) of the top candidate SNPs previously proposed. (B) The top panel of “A” indicates the the derived allele frequency (DAF) of 10 top SNPs on chromosome 5 and 7 proposed in Table 3 in study of Moradi et al.. The s553221.1 locus on chromosome 5 in their study was not included because it did not pass the quality control in this study. (B) The middle panel of “A” shows the DAF of four SNPs corresponding to BMP2 and two SNPs corresponding to VRTN gene proposed in Table 2 in study of Moioli et al.. The s73063.1 locus annotated to VRTN was not was not included because it did not pass the quality control in this study. (C) The bottom panel of “A” represents the DAF of SNPs annotated to the key genes proposed in Table 1 and Table 2 in study of Yuan et al. The OAR4_73050615.1 locus is from Table 1 and other loci are from Table 2 in their study. [file 12864_2020_7210_MOESM10_ESM.pdf]

Figure S4

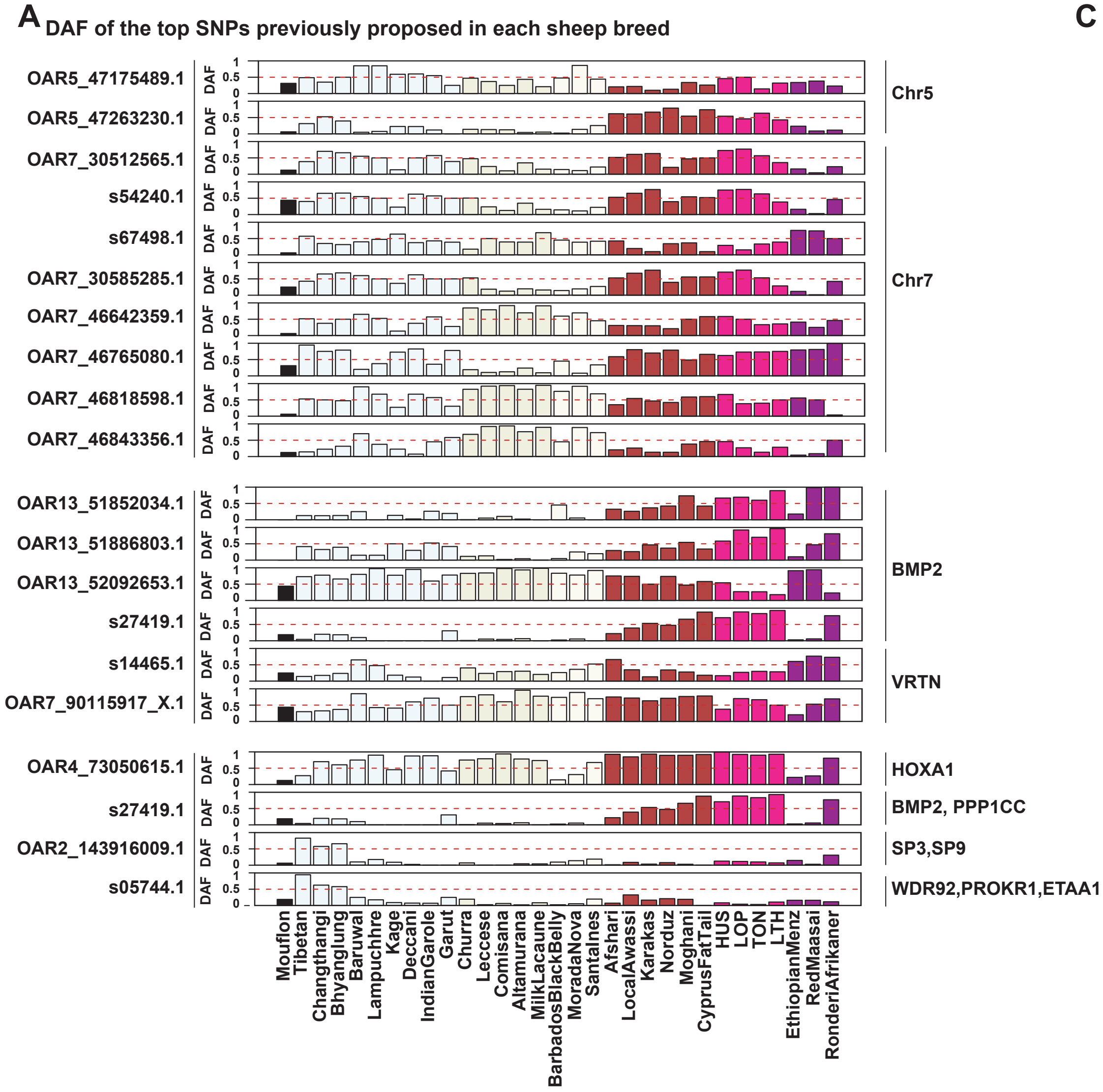

C Snapshot of Table 2 in study of Moioli et al.

Table 2. Genomic regions encoding putative candidate genes of the fat tail phenotype

Chromosome	Marker	Position on Chromosome v3.1[1]	Gene			
			Symbol	Start	Stop	Accession no.
2	OAR2_115949202	107805210	–			
	OAR2_116017982	107866879				
	OAR2_116054738	107911874				
6	OAR6_44329387	39650297	SLIT2	39691904	40093085	EF627036
	OAR6_44345133	39662841				
7	s14465.1	82694743	VRTN	82585586	82587626	XM_004010786
	OAR7_90115917_X.1	82749607				
	s73063.1	82851545				
10	s68983.1	30618253	ALOX5AP	30365140	30388562	XM_004012141
	s12004.1	30674002				
10	s27955.1	35596332	FGF9	35583697	35610038	XM_004012161
	s26969.1	35629123				
13	OAR13_51852034.1	48623826	BMP2	48460133	48473737	XM_004014353
	OAR13_51886803.1	48657027				
	OAR13_52092653.1	48832966				
	s27419.1	48968332				

B Snapshot of Table 3 in study of Moradi et al.

Table 3 The table of significant test for Zel-Lori Bakhtiri and ovine HapMap data set.

SNP_Name	Chr	Coordinate	Zel-Lori Bakhtiri data set		HapMap data set		Fisher's combined
			windowed F_{ST}	P-value	windowed F_{ST}	P-value	P-value
OAR2_55861669.1	2	55861669	0.24	0.00051	0.06	0.08192	0.0004593
s63030.1	2	55922894	0.25	0.00042	0.06	0.07696	0.0003646
s20468.1	2	56248983	0.24	0.00048	0.10	0.01297	0.0000814*
s58048.1	2	56288982	0.23	0.00053	0.10	0.01303	0.0000886*
OAR2_56300406.1	2	56300406	0.22	0.00064	0.10	0.01441	0.0001157
s42805.1	2	73631109	0.23	0.00055	0.02	0.52322	0.0026316
OAR2_73687773.1	2	73687773	0.25	0.00044	0.02	0.46291	0.0019329
OAR2_73744625.1	2	73744625	0.29	0.00024	0.02	0.42839	0.0010538
OAR2_73784818.1	2	73784818	0.26	0.00040	0.03	0.38720	0.0014986
s35724.1	3	146673736	0.22	0.00066	0.01	0.71086	0.0040613
s55322.1	5	47149400	0.26	0.00037	0.13	0.00505	0.0000267*
OAR5_47175489.1	5	47175489	0.28	0.00029	0.17	0.00140	0.0000063*
OAR5_47263230.1	5	47263230	0.29	0.00022	0.12	0.00669	0.0000212*
OAR7_30512565.1	7	30512565	0.22	0.00068	0.02	0.45742	0.0028276
s54240.1	7	30525058	0.29	0.00026	0.03	0.25759	0.0007199
s67498.1	7	30572812	0.27	0.00035	0.04	0.19337	0.0007205
OAR7_30585285.1	7	30585285	0.22	0.00062	0.04	0.17072	0.0010675
OAR7_46642359.1	7	46642359	0.27	0.00033	0.15	0.00282	0.0000138*
OAR7_46765080.1	7	46765080	0.34	0.00015	0.16	0.00177	0.0000044*
OAR7_46818598.1	7	46818598	0.28	0.00031	0.17	0.00161	0.0000077*
OAR7_46843356.1	7	46843356	0.25	0.00046	0.13	0.00476	0.0000308*
OARX_58621412.1	X	58621412	0.22	0.00057	0.05	0.09020	0.0005604
OARX_59194976.1	X	59194976	0.30	0.00020	0.12	0.00647	0.0000186*
OARX_59257971.1	X	59257971	0.38	0.00013	0.19	0.00088	0.0000020*
OARX_59327581.1	X	59327581	0.44	0.00009	0.21	0.00048	0.0000008*
OARX_59383635.1	X	59383635	0.55	0.00004	0.27	0.00019	0.0000002*
OARX_59571364.1	X	59571364	0.58	0.00002	0.27	0.00017	0.0000001*
OARX_59578440.1	X	59578440	0.49	0.00007	0.22	0.00038	0.0000005*
OARX_59912586.1	X	59912586	0.38	0.00011	0.17	0.00144	0.0000026*
s38079.1	X	60149273	0.34	0.00018	0.16	0.00179	0.0000050*
OARX_60238540.1	X	60238540	0.21	0.00070	0.07	0.03783	0.0003069
s31917.1	X	61452816	0.22	0.00059	0.04	0.19923	0.0011873

Position, windowed F_{ST}, empirical P-values and Fisher's combined P-values of observed peaks in Zel-Lori Bakhtiri data set (windowed F_{ST} > 0.20) and comparison to independent experiment using Ovine HapMap data

* Significant *P*-Value < 0.0001

D Snapshot of Table 1 and Table 2 in study of Yuan et al.

Table 1 Candidate regions and genes identified by hapFLK.

Region	Chr.	Start	Stop	Size (Mb)	Peak SNP	*P*-value	No. of genes	Peak gene	Genes
1	2	133.2	137.10	3.90	OAR2_1439416009.1	4.11E-06	23	*CDCA7*	*ATP5G3, ATF2, RPL17, CHN1, CHRNA1, GPR155, SCRN3, CIR1, SP9OLA1, SP3, CDCA7, MLTK, RAPGEF4, AIMP1, PDK1, ITGA6, DLX2, DLX1, METAP1D, HAT1, SLC25A12, DYNC1I2, CYBRD1*
2	3	37.17	43.07	5.90	s05744.1	8.99E-5	37	*WDR92*	*LCLAT1, CAPN13, GALNT14, CAPN14, EHD3, SNRPG, PCYOX1, GLRX5, TIA1, CFDP2, PCBP1, ASPRV1, MXD1, SNRNP27, GMCL1, BMP2K, NFU1, GFPT1, HERC5, ANTXR1, GKN1, POL, GKN2, BMP10, ARHGAP25, PROKR1, APLF, FBXO48, PLEK, CNRIP1, PPP3R1, PNO1, WDR92, ETAA1, MEIS2, GSR, SPRED2*
3	4	66.84	70.32	3.48	OAR4_73050615.1	2.76E-05	29	*HOXA1, SKAP2*	*CPVL, TRIL, CREB5, RPS15A, CREB5, JAZF1, TAX1BP1, RAN, HIBADH, RPS18, EVX1, HOXA11, HOXA10, HOXA9, HOXA7, HOXA6, HOXA5, HOXA3, HOXA4, HOXA2, HOXA1, SKAP2, SNX10, CBX3, HNRNPA2B1, NFE2L3, SLC30A9, SLC30A9, STK32B*
4	10	26.82	32.28	5.6	OAR10_29511510.1	1.61E-14	33	*RXFP2*	*SLC25A5, RFC3, CFDP2, STARD13, KL, PDS5B, N4BP2L2, N4BP2L1, BRCA2, ZAR1L, FRY, EEF1A1, RXFP2, B3GALTL, HSPH1, C13ORF26, ALOX5AP, USPL1, HMGB1, HMGB1, KATNAL1, UBL3, SLC7A1, MTUS2, MTUS2, SLC46A3, POMP, FLT1, PAN3, FLT3, PRHOXNB, CDX2, GSX1, POLR1D, HSPE1, LNX2, MTIF3, GTF3A*
5	13	44.11	52.89	8.78	OAR13_51852034.1	2.64E-13	67	*BMP2*、*PPP1CC*	*PFK, ADARB2, F5, WDR37, IDI1, GTPBP4, LARP4B, DIP2C, CFDP2, ZMYND11, PRNP, PRND, RASSF2, SLC23A2, PCNA, CDS2, PROKR2, GPCPD1, CHGB, TRMT6, MCM8, CRLS1, LRRN4, FERMT1, BMP2, PPP1CC, MUTED, UBA52, HAO1, ADRA1D, SMOX, RNF24, PANK2, MAVS, C20ORF29, CDC25B, CENPB, SPEF1, HSPA12B, SIGLEC1, ADAM33, ATRN, WDR45L, C20ORF194, SLC4A11, ITPA, DDRGK1, PROSAPIP1, PROSAPIP1, FASTKD5, UBOX5, PTPRA, VPS16, FAM113A, C20ORF141, CPXM1, IDH3B, NOP56, TMC2, SNRPB, TGM6, TGM3, STK35, PDYN, SIRPA, SIRPA, FAM108C1*
6	15	2.50	5.77	3.28	OAR15_2999185.1	2.25E-06	11	*PDGFD*	*MRPL32, CASP13, DDI1, PDGFD, DYNC2H1, DCUN1D5, MMP13, MMP12, MMP3, MMP1, MMP27*
7	22	38.22	41.73	3.51	s02448.1	1.47E-4	22	*FGFR2*	*FAM45A, SFXN4, PRDX3, GRK5, TIAL1, BAG3, INPP5F, MCMBP, SEC23IP, PPAPDC1A, WDR11, IL11, FGFR2, ATE1, ZNRD1AS, TACC2, TACC2, BTBD16, HTRA1, DMBT1, DMBT1, IKZFPSTK*

Table 2 Candidate regions and genes identified by F_{ST}.

Region	Chr	Position (Mb)	Peak SNP (F_{ST})	Top SNP	Peak gene	Genes
1	1	14.69–14.93	OAR1_14614448.1 (0.35)	1	*SMAP2*	*COL9A2, SMAP2, ZNF642, DEM1, ZNF684, GTF2F2, RIMS3*
2	1	94.84–95.27	OAR1_100921673.1 (0.46)	2	–	–
3	1	215.05–215.35	OAR1_232334848.1(0.38)	1	*PHC3*	*SKIL, PRKCI, RPL17, PHC3, GPR160*
4	2	134.30–135.45	OAR2_143916009.1 (0.49)	4	*SP3*	*CHN1, CHRNA1, GPR155, SCRN3, CIR1, SP9, OLA1, SP3*
5	3	39.58–40.91	s05744.1 (0.65)	5	*WDR92*	*PROKR1, APLF, FBXO48, PLEK, CNRIP1, PPP3R1, PNO1, WDR92, ETAA1*
6	3	78.37–78.52	s26507.1 (0.37)	1	–	–
7	3	93.33–93.98	s64982.1 (0.49)	2	*DYSF*	*DYSF, CYP26B1*
8	3	154.16–154.26	OAR3_165009241.1 (0.47)	1	*MSRB3*	*MSRB3*
9	4	48.42–48.95	OAR4_51489408.1 (0.46)	1	*BCAP29*	*COG5, GPR22, DUS4L, BCAP29, SLC26A4, SLC26A3, DLD*
10	4	68.36–69.21	OAR4_72720594.1 (0.57)	3	*RPS18*	*TAX1BP1, RAN, HIBADH, RPS18, VX1, HOXA11, HOXA10, HOXA9, HOXA7, HOXA6, HOXA5, HOXA3, HOXA4, HOXA2, HOXA1, SKAP2*
11	4	87.51–87.73	OAR4_93067608.1 (0.41)	1	*SLC13A1*	*SLC13A1, IQUB*
12	6	36.28–36.66	OAR6_40780034.1 (0.39)	1	*ABCG2*	*HERC6, PPM1K, ABCG2, PKD2*
13	6	96.61–96.84	OAR6_106396859.1 (0.39)	1	*VAMP714*	*VAMP714, HNRNPD, NAP1L1, HNRPDL, ENOPH1*
14	7	96.61–96.84	s55494.1 (0.46)	1	*VSX2*	*QTSA11302, ALDH6A1, LIN52, VSX2, ABCD4, VRTN, TMEM90A, NPC2, ISCA, LTBP2*
15	8	56.03–56.29	s12519.1 (0.38)	1	–	–
16	9	1.30–1.46	OAR9_1100990.1 (0.41)	1	–	–
17	9	93.46–93.80	OAR9_99601991.1 (0.39)	1	*ENPP2*	*ENPP2, TAF2, DSCC1*
18	10	29.24–29.61	OAR10_29469450.1 (0.71)	3	*RXFP2*	*FRY, EEF1A1, RXFP2, B3GALTL*
19	11	18.24–18.58	OAR11_18701428.1(0.44)	3	*NF1*	*NF1, EVI2A, EVI2B, OMG*
20	13	47.77–49.21	s27419.1 (0.65)	5	*PPP1CC*	*BMP2, PPP1CC, MUTED, UBA52*
21	13	62.77–63.46	s12884.1 (0.38)	1	*RALY*	*RALY, EIF2S2, ASIP, AHCY, AHCY, ITCH*
22	15	3.24–3.99	OAR15_3091174.1 (0.61)	2	*PDGFD*	*PDGFD, DDI1*
23	16	16.99–17.14	OAR16_18729547.1 (0.37)	1	*IPO11*	*IPO11, DIMT1L, KIF2A*
24	22	36.65–36.90	s19503.1 (0.36)	1	*EMX2*	*PDZD8, EMX2*
25	25	7.20–7.69	s25195.1 (0.40)	2	*EIF2S2*	*SLC35F3, C1ORF31, TARBP1, EIF2S2, IRF2BP2*
26	25	38.63–38.83	s21601.1 (0.38)	1	*GHITM,*	*GHITM, CDHR1, LRIT2, LRIT1*
